# A Dynamic and Combinatorial Histone Code Drives Malaria Parasite Asexual and Sexual Development

**DOI:** 10.1016/j.mcpro.2022.100199

**Published:** 2022-01-17

**Authors:** Hilde von Grüning, Mariel Coradin, Mariel R. Mendoza, Janette Reader, Simone Sidoli, Benjamin A. Garcia, Lyn-Marié Birkholtz

**Affiliations:** 1 Department of Biochemistry, Genetics and Microbiology, University of Pretoria, Pretoria, South Africa; 2 Institute for Sustainable Malaria Control, University of Pretoria, Pretoria, South Africa; 3 Department of Biochemistry and Biophysics, Epigenetics Institute, Perelman School of Medicine, University of Pennsylvania, Philadelphia, Pennsylvania, USA; 4 Department of Biochemistry, Albert Einstein College of Medicine, Bronx, New York, USA

**Keywords:** gametocyte, histone code, malaria, middle-down mass spectrometry proteomics, parasite, *Plasmodium falciparum*, posttranslational modification, PTM, ADA2, Transcriptional adaptor protein 2, AP2, Apetala2/ethylene response factor protein family, CARM1, Coactivator-associated arginine methyltransferase 1, CBP, CREB-binding protein, CHD1, Helicase-DNA-binding protein 1, GCN5, General control nonrepressed 5 protein acetyltransferase, GluC, Glucosidase C protease, HP1, Heterochromatin protein 1, iBAQ, Intensity-based absolute quantification, IS, Interplay score, ISWI, Imitation switch chromatin remodeling protein family, MS, mass spectrometry, NAPS, Nucleosome assembly protein, PHD, Plant homeodomain, PTM, Posttranslational modification, SAGA, Spt-Ada-Gcn5 acetyltransferase, Snf2, Sucrose nonfermentable

## Abstract

Histone posttranslational modifications (PTMs) frequently co-occur on the same chromatin domains or even in the same molecule. It is now established that these “histone codes” are the result of cross talk between enzymes that catalyze multiple PTMs with univocal readout as compared with these PTMs in isolation. Here, we performed a comprehensive identification and quantification of histone codes of the malaria parasite, *Plasmodium falciparum*. We used advanced quantitative middle-down proteomics to identify combinations of PTMs in both the proliferative, asexual stages and transmissible, sexual gametocyte stages of *P. falciparum*. We provide an updated, high-resolution compendium of 77 PTMs on H3 and H3.3, of which 34 are newly identified in *P. falciparum*. Coexisting PTMs with unique stage distinctions were identified, indicating that many of these combinatorial PTMs are associated with specific stages of the parasite life cycle. We focused on the code H3R17me2K18acK23ac for its unique presence in mature gametocytes; chromatin proteomics identified a gametocyte-specific SAGA-like effector complex including the transcription factor AP2-G2, which we tied to this specific histone code, as involved in regulating gene expression in mature gametocytes. Ultimately, this study unveils previously undiscovered histone PTMs and their functional relationship with coexisting partners. These results highlight that investigating chromatin regulation in the parasite using single histone PTM assays might overlook higher-order gene regulation for distinct proliferation and differentiation processes.

## Highlights

• First middle-down chromatin proteomics compendium of the malaria parasite, *Plasmodium falciparum.*
• Novel histone PTMs (including arginine methylation) in both asexual parasites and transmissible gametocytes.
• Histone PTM cross talk is dynamic life cycle stage stratified.
• Gametocytes rely on histone PTM connectivity to allow onward transmission.
• AP2-G2 is an important effector of H3K18acK23ac in mature gametocytes.

## Graphical Abstract

**Figure:**
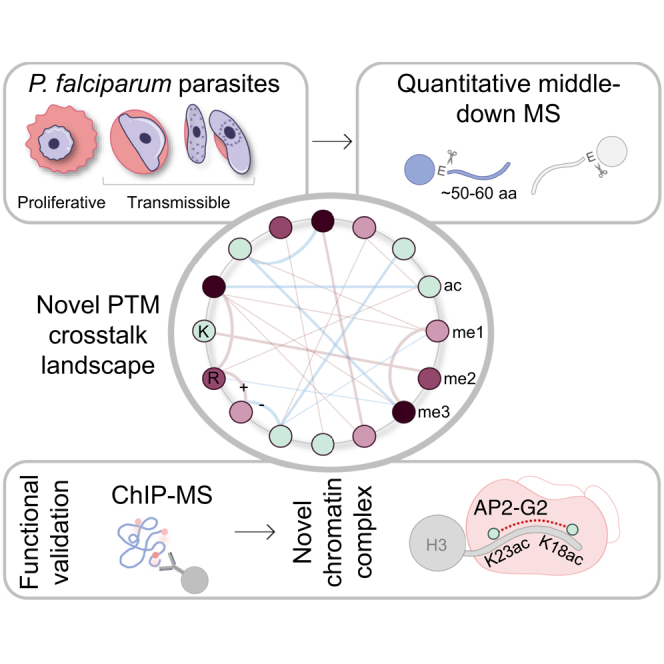

## In Brief

The complex combinatorial histone code of the malaria parasite is revealed through advanced, quantitative middle-down proteomics. Cross talk between histone PTMs (including novel PTMs) is dynamic and stage specific and includes arginine methylation. Chromatin proteomics show that the transcription factor AP2-G2 interacts with the combination of H3K18acK23ac to drive mature gametocyte transmissibility. The connectivity of the histone code in these parasites ultimately points toward a higher order of gene regulation involved in essential development processes than previously thought.

Histone N-terminal tails are reversibly modified by an array of covalent histone posttranslational modifications (PTMs). These alter chromatin structure to fine-tune gene expression in most eukaryotes, resulting in changes in cell fate. Although the contribution of individual histone PTMs in specific biological processes are well described (1, 2), there is growing evidence to indicate that histone PTMs not only function individually but also act in a concerted manner to direct transcriptional programs according to the cell’s immediate needs. The outcome of such coordination ultimately defines a cell’s fate and function (3), such as to proliferate (4, 5), differentiate (6, 7), or become quiescent (8, 9). This association between histone PTMs that work in coordination has been postulated to constitute a functionally relevant, unique pattern or “histone code” (10, 11).

As an example of the importance of histone PTM cross talk, the presence of histone H3, serine 10 phosphorylation (H3S10ph) impairs the binding of the effector protein heterochromatin protein 1 (HP1) to the well-known repressive PTM, H3K9me3 (12). This blocks cellular differentiation in mouse embryonic stem cells (13, 14). Such drastic changes in gene regulation and cellular fate can also be effected by combinations of PTMs that include typically less abundant PTMs, *e.g.*, H3K9ac and H3K14ac affect H3R8me2 (15, 16).

*Plasmodium falciparum* is the causative agent of severe malaria in humans and undergoes rapid rounds of cell division during its asexual replication to proliferate every ∼48 h. A small percentage of asexual parasites differentiate to gametocytes through five distinct stages of development (stages I–V) over ∼14 days in *P. falciparum*, after which mature male and female stage V gametocytes can be transmitted to the mosquito vector (17, 18). The parasite’s chromatin organization fluctuates between mostly euchromatic in asexual parasites, characterized by transcriptionally permissive PTMs of H3K9ac and H3K4me3 (19, 20, 21), and more heterochromatic states during gametocytogenesis, marked with H3K9me3, H4K20me3, and extended HP1 occupancy (22, 23). However, nuanced distinctions exist between the different developmental stages during gametocytogenesis and underscore the transcriptional differences between the early- (stage II/III) and late-stage (stage IV/V) gametocytes (24). Typical heterochromatic PTMs such as H3K27me3 (facultative heterochromatin), H3K9me3 (constitutive heterochromatin), and H3K36me3 are exclusive to the immature, early stages in *P. falciparum* (22, 25), whereas more mature stages do contain euchromatic PTMs (H3K4me3 and H4K8ac) in preparation for onward transmission and gamete formation (22).

Evidence of histone PTM combinations in *P. falciparum* is sparse but includes methylation of H3K4 or H3K9 by the histone lysine N-methyltransferase (su(var)-3–9, enhancer-of-zeste, trithorax) domain-containing protein 7 (SET7), only in the presence of already acetylated H3K14 (26). H4K8ac, as a likely regulator of parasite proliferation in asexual parasites (27), is also found in combination with H4K5ac, H4K12ac, and H4K16ac as a result of the acetyltransferase activity of MYST (28). Quantitative chromatin proteomics alluded to the presence of additional coexisting histone PTMs in *P. falciparum* parasites, with prominent stage specificity and increased presence during gametocytogenesis (22, 29). Several of the individual histone PTMs have been demonstrated to be essential to various biological processes, through gene knockout or chemical perturbation of histone “writer” and “eraser” enzymes (30, 31).

The majority of coexisting histone PTM pairs are typically identified with a peptide-centric proteomics pipeline, frequently named “bottom-up.” Histones are digested with trypsin or other enzymes that generate relatively short peptides before mass spectrometry (MS) analysis. With this workflow, co-occurrences between histone PTMs can be identified only for those that localize nearby in the amino acid sequence. However, this proteomic approach is not suitable for distal co-occurring PTMs. As such, distinctively modified peptides and co-occurring PTMs cannot be accurately identified and quantified. The development of “middle-down” MS has advanced proteomics and allowed the complexity of PTM combinations to be investigated (32, 33). Although technically challenging, middle-down MS allows evaluation of longer histone tails of ∼50 to 60 residues to identify and quantify hundreds of combinatorial PTMs accurately and simultaneously. With this powerful approach, system-level cross talk of multiple, interacting coexisting PTMs defined clear, combinatorial histone codes in nematodes (34), mammalian cells (4, 35, 36, 37), cells undergoing epithelial to mesenchymal transition (37, 38, 39, 40, 41), and stem cell reprogramming (42).

Here, we present the systems-level identification and characterization of the combinatorial histone code of *P. falciparum* parasites, which was generated using quantitative, middle-down proteomics. We identify a comprehensive histone code that is dynamic and has distinct fingerprints in different life cycle stages, implying refined functions to allow different biological outcomes associated with parasite pathology and survival. Several coexisting PTMs are involved in direct cross talk, indicative of coordinated function, particularly in gametocytes, with the code in immature gametocytes being most connected and mature gametocytes using unique combinations. This is exemplified by the functional association of H3K18acK23ac that interacts with a unique reader complex in mature gametocytes to enable strategy-specific gene expression. With this first, comprehensive report of a combinatorial histone code in a eukaryotic parasite, we show that *P. falciparum* relies on dynamic interactions between histone PTMs for development and differentiation. These data could serve as a model for the importance of combinatorial histone PTMs in pathogenesis in protista.

## Experimental Procedures

### *In Vitro* Cultivation of *P. falciparum* Asexual Parasites and Gametocytes

All *in vitro* experiments involving human blood donors and human malaria parasites hold ethics approval from the University of Pretoria Research Ethics Committee, Health Sciences Faculty (NAS332/2019). This work abides by the Declaration of Helsinki principles. Intraerythrocytic *P. falciparum* parasites (NF54 strain, drug sensitive) were cultivated in fresh human erythrocytes (either A⁺ or O+) in RPMI-1640 culture medium supplemented with 25 mM Hepes (pH 7.5, Sigma-Aldrich), 0.2 mM hypoxanthine (Sigma-Aldrich), 0.024 μg/μl gentamycin (Hyclone), 5 μg/μl Albumax II (Invitrogen), 23.81 mM sodium bicarbonate (Sigma-Aldrich) and 0.2% w/v D-glucose. Cultures were maintained with daily media change and fresh erythrocyte supplementation at 5% hematocrit, 2% parasitemia under hypoxic conditions (5% O₂, 5% CO₂, 90% N₂) with moderate shaking at 37 °C. Parasites were synchronized to more than 90% rings stages with D-sorbitol. Gametocytogenesis production was initiated at 0.5% parasitemia and a 6% hematocrit in a glucose-free medium under hypoxic gaseous (5% O₂, 5% CO₂, 90% N₂) conditions at 37 °C without shaking (43).

### Histone Enrichment From *P. falciparum* Parasites

Histones were enriched using a protocol described (44) and adapted (22). The asexual parasites and gametocytes were freed from erythrocytes using 0.06% w/v saponin in phosphate-buffered saline (PBS) and centrifuged at 12,000*g* for 10 min. The resulting erythrocyte pellet was repeatedly washed three times by suspending the pellet in PBS followed by centrifugation. Nuclei were liberated from *P. falciparum* parasites using a hypotonic buffer containing 10 mM Tris-HCl (pH 8.0), 3 mM MgCl₂, 0.2% v/v Nonidet P-40, 0.25 M sucrose, and a cocktail of EDTA-free cocktail of protease inhibitors. This hypotonic buffer wash stage was repeated twice with the mixture centrifuged at 500*g* and 4 °C for 10 min. The chromatin pellet was next homogenized in a hypotonic buffer lacking Nonidet P-40 (10 mM Tris-HCl, pH 8.0, 0.8 M NaCl, 1 mM EDTA [including protease inhibitor cocktail]) and incubated on ice for 10 min. Histones were subsequently extracted from chromatin and enriched through incubation with 0.25 M hydrochloric acid and then rotated for 1 h at 4 °C. The histone-containing supernatant was combined with an equal volume of 20% trichloroacetic acid then pelleted after 15 min on ice. The histone-enriched pellet was washed with acetone, air dried, and reconstituted using dddH_2_O. A SpeedVac concentrator was used to dry all the samples (SC100, Savant), which were stored at −80 °C.

### Middle-down Proteomics for Identification of Combinatorial Histone PTMs

Middle-down MS was performed according to Coradin *et al.* (2020) with a few modifications (34, 41, 45). Histones (15–50 μg) were suspended in 5 mM ammonium acetate to a final concentration of 0.5 μg/μl (pH 4.0). Glucosidase C protease (GluC) endoprotease was diluted to 0.2 μg/μl in the same buffer and added to the histone samples a final concentration of 1:20 GluC enzyme:histone. Histone samples were incubated overnight at room temperature, and digestion was blocked by the addition of 1% formic acid. Trifluoroacetic acid (0.1%) was added to the digested peptides and desalted using StageTips packed with a solid phase C_18_ column disk (3M Empore) with porous graphitic carbon suspended in 100% acetonitrile on top and washed with 0.1% trifluoroacetic acid. Peptides were eluted by the addition of 70% acetonitrile and 0.1% trifluoroacetic acid elution buffer. Histone protein samples were dried and suspended to a concentration of ∼2 μg/μl in the sample buffer consisting of 60% acetonitrile, 20 mM propionic acid, and ethylenediamine.

Separation of digested histone peptides was performed by using nanoliter flow liquid chromatography using an EASY-nLC nanoHPLC (Thermo Scientific) equipped with an analytical weak cation exchange–hydrophilic interaction liquid chromatographic resin (PolycatA, PolyLC) column (75 μm ID, 15 cm length, 1.7 μm diameter, and 1000 Å porosity). Buffer A was prepared with 70% acetonitrile and 20 mM propionic acid to adjust the pH to 6.0. Buffer B consisted of 0.1% formic acid in LC-MS grade H_2_O. The HPLC gradient was set up for a nonlinear gradient: 0% to 70% of buffer B for 2 min followed with 72% to 85% buffer B for more than 90 min. The gradient was on hold for 5 min at 95% buffer B to wash the column before sequential sample loading. To minimize sample carryover, blank injections were run between different samples for at least 20 min with high (80%–95%) buffer B.

MS detection was performed using an Orbitrap Fusion mass spectrometer (Thermo Scientific) operated in a data-dependent acquisition mode and high mass resolution mode for both MS1 events and MS2 scans for each of the three stages investigated in 2 to 3 independent biological experiments. The full mass spectrum scan was set at 665 to 705 *m/z*, as this is the range of the most intense charge states (+8) for histone H3 polypeptides. A charge filter was added to include +8 charge states targeted for fragmentation. The ion transfer tube temperature was set to 300 °C and the spray voltage to 2.3 kV. To fragment peptides while retaining PTM information, electron transfer dissociation was performed (46, 47) at a resolution setting of 120,000 (MS1) and 30,000 (MS2). The electron transfer dissociation reaction time was 20 ms for polypeptides with +8 charge states. For high-resolution spectrums, three microscans were averaged.

### Middle-down Proteomics Data Analysis

Spectra were analyzed with Proteome Discoverer (v. 2.4, Thermo Scientific), including Xtract (Thermo) for spectra deconvolution. Three UniProt entries of histone H3 sequences for *P. falciparum* (cross checked with histone sequences obtained from PlasmoDB resource [https://plasmodb.org/, version 43, released 25 April 2019]) were searched using Mascot (v. 2.5, Matrix Science). During individual spectra identification, spectra were filtered on multiple layers: (1) deconvolution discarded all MS/MS ions with no identified charge state; (2) spectral identification was performed using the Mascot algorithm without filters; (3) the Mascot output file was filtered using a custom software named ProteoformQuant (48). This software eliminates all PTM identifications that are not unambiguously supported by MS/MS fragment ions, *e.g.*, a peptide with a modification that could be placed on more than one residue would not pass the filter; (4) for those identifications that pass the ProteoformQuant filter, all mixed MS/MS spectra without unambiguous fragment ions for differential quantification of isobaric peptides are discarded (explained in (49)).

No missed or nonspecific cleavages were considered; only fully digested N-terminal tails were considered. Endoproteinase GluC was used to generate peptides, which is a serine protease that cleaves peptide bonds at the C terminus of glutamic acid residues. Files were searched with the following dynamic modifications: acetylation (K), phosphorylation (ST), mono and dimethylation (RK), and trimethylation (K). No fixed modifications were considered. The mass tolerance was set to 2.1 Da for precursor ions and 0.01 Da for fragment ions. IsoScale (http://middle-down.github.io/Software/) was used to confidently identify and quantify modified peptides. Only *c/z* fragment ions were allowed. PTMs were accepted only if there were at least one site determining ions on both sides of the assigned PTM. Estimation of false discovery rate (FDR) is not applicable for middle-down proteomics as peptides are too long to have any decoy match. A list of all peptide sequences identified and sites of modifications are contained in Appendix 1 (ambiguous matches are discarded by ProteoformQuant). Peptide identification scores are not applicable.

Interplay scores between bivalent histone PTMs were calculated according to the following equation:

$$Interplay_{PTM1PTM2}=\;\log\;2\;\frac{F_{PTM1PTM2}}{F_{PTM1}\;\times \;F_{PTM2}}$$

where *F*_*PTM*1*PTM*2_ is the observed relative abundance (*F*) of the combination divided by the predicted relative abundance of the combination, calculated by multiplication of the relative abundances of the individual histone PTMs. The relative abundance of the individual PTMs was calculated by summing the relative abundances of all combinatorial peptides containing the particular PTM. If the interplay score is positive, the PTMs are likely codependent or positively related; a negative interplay score indicates that the PTMs are mutually exclusive. Ring plots were visualized using Cytoscape (v. 3.8.2). Other plots were created using GraphPad Prism 9 and R (R Studio v. 4.0.3).

### Chromatin Immunoprecipitation for Analysis of Histone PTM-associated Proteins

To identify proteins that associate to individual histone PTMs that occur in combination, chromatin immunoprecipitation (ChIP)-MS was performed using an adapted protocol (50). A minimum number of isolated parasites (10^9^ cells/ml) was cross-linked with 1% formaldehyde and subsequently quenched with 125 mM glycine. Cross-linked parasite nuclei were suspended in cold lysis buffer (10 mM Hepes pH 7.9, 10 mM KCl, 0.1 mM EDTA pH 8.0, 0.1 mM EGTA pH 8.0, 1 mM DTT, and EDTA-free protease inhibitor cocktail) and transferred to a prechilled dounce homogenizer. NP40 was added to a final concentration of 0.25%, and parasites were subsequently lysed with dounce B for ∼100 strokes. Sonication shearing buffer (a cocktail of protease inhibitors, 1% SDS, 50 mM Tris-HCl (pH 8.0), 10 mM EDTA, and 100 mM NaCl) was added to the nuclei pellet and sonicated with the BioRuptor UCD-200 (Diagenode) for 25 cycles at high power and 30-s intervals. Cross-links were reversed by incubating the input sample overnight at 65 °C. The input sample was suspended to a final volume of 200 μl with ChIP dilution buffer (0.01% SDS, 1% Triton X-100, 1.2 mM EDTA, 16.7 mM Tris-HCl, pH 8.0, 150 mM NaCl, and a cocktail of protease inhibitors). To dilute the SDS in the dilution buffer, Tris-EDTA buffer (1 M Tris, pH 8.0 and 0.5 M EDTA, pH 8.0) was added with RNase to a final concentration of 0.2 μg/μl and incubated for 1 h at 37 °C. The chromatin soup was incubated with 1 μg of either anti-H3K18ac or anti-H3K23ac, rotating at 4 °C overnight. Protein G magnetic Dynabeads (Invitrogen by Thermo Fisher Scientific) was added and incubated for 2 h rotating at 4 °C. A total of 20% of the eluted chromatin was then retained as the input. The remaining eluted material was then used for the Western blot validation of combination histone PTM. The bead–chromatin complex was washed extensively with each of the following buffers with the aspiration of the preceding buffer before the addition of the next buffer: low-salt immune complex wash buffer (0.1% SDS, 1% Triton X-100, 2 mM EDTA, 20 mM Tris-HCl pH 8.1, 150 mM NaCl), high-salt immune complex wash buffer (0.1% SDS, 1% Triton X-100, 2 mM EDTA, 20 mM Tris-HCl pH 8.1, 500 mM NaCl), LiCl immune complex wash buffer (0.25 M LiCl, 1% NP-40, 1% deoxycholate, 1 mM EDTA, 10 mM Tris-HCl pH 8.1), and TE Buffer (10 mM Tris-HCl pH 8.0, 1 mM EDTA pH 8.0). The beads were suspended in fresh ChIP elution buffer (0.1 M NaHCO3, 1% SDS), and the supernatant was collected.

### Quantitative Mass Spectrometry of Histone PTM-associated Proteins Identified From the ChIP

Briefly, protein samples were mixed with an equal volume of methanol:chloroform (3:1 ratio), followed by a brief vortex step and centrifugation at 4 °C. Samples were washed twice with methanol followed by centrifugation at 17,000*g* for 3 min and complete removal of methanol:chloroform. The resulting precipitated proteins were dried at room temperature. All samples were dissolved in a solution containing 6 M urea, 2 M thiourea, and 50 mM ammonium bicarbonate, pH 7 to 8. Samples were subjected to reduction, alkylation, and digestion in preparation for MS as follows. Samples were incubated at room temperature for 1 h in 5 mM DTT to reduce disulfide bonds, cysteine residues alkylated in 50 mM iodoacetamide, and subjected to Lys-C (1–1.5 μg) and trypsin (10 μg) in-solution digestion at room temperature for 3 h. Samples were then diluted with 50 mM ammonium bicarbonate to lower the urea and DTT concentrations in solution to prevent trypsin inactivation. The samples were sonicated (Sonic Dismembrator Model 100, Fischer Scientific) for three cycles of 10-s continuous sonication (output 2) followed by 10-s resting on ice to dissolve the pellets completely. Cysteine residues were alkylated by incubation with 40 mM iodoacetamide. This was followed by overnight digestion with 10 μg trypsin at room temperature. The pH of the trypsin digested samples was adjusted with ammonium hydroxide to ∼pH 8 to 8.3 followed by centrifugation at 17,000*g* for 1 min. StageTip (STop And Go Extraction Tip) cleanup in combination with protein fractionation was performed for all the samples using a dual resin approach, where both Empore C18 disks (3 M) and OligoTM R3 reversed-phase resins were used in combination. This was done to remove unwanted contaminants before MS and to fractionate the samples to ensure optimal protein detection. The column was equilibrated with 1 mM ammonium bicarbonate (pH 8.0), after which the supernatant of each sample was added to the dual resin StageTip and pushed through the column using a syringe (dropwise). All samples were dried down completely under vacuum (SpeedVac concentrator). In preparation for the MS run, 0.1% formic acid was added to the samples followed by ultrasonic bath sonication for 10 min at 4 °C. The samples were centrifuged at 17,000*g* for 10 min, and the supernatant was loaded onto a Dionex LC system (Thermo Fisher Scientific), coupled online with a Q-Exactive HF mass spectrometer (Thermo Scientific). Peptides were loaded into a picofrit 20-cm-long fused silica capillary column (75 μm inner diameter) packed in-house with reversed-phase Repro-Sil Pur C18-AQ 3-μm resin. A gradient of 105 min was set for peptide elution from 2% to 28% buffer B (100% acetonitrile/0.1% formic acid), followed by a gradient from 28% to 80% buffer B in 5 min and an isocratic 80% B for 10 min. The flow rate was set at 300 nl/min. The MS method was set up in a data-dependent acquisition mode. The full MS scan was performed at 70,000 resolutions (full width at half maximum at 200 *m/z*) in the *m/z* range 350 to 1200 and an automatic gain control target of 10e6. Tandem MS (MS/MS) was performed at a resolution of 17,500 with a collision-induced dissociation collision energy set to 20; an automatic gain control target of 5 × 10e4; a maximum injection time of 100 ms; a loop count of 12; an intensity threshold for signal selection at 10e4, including charge states 2 to 4; and a dynamic exclusion set to 45 s.

### Chromatin Proteomic Profiling Data Analysis

MS raw files were analyzed by MaxQuant software version 1.5.2.8. MS/MS spectra were searched against the *P. falciparum* UniProt FASTA database, containing 33,655 entries. Sequencing grade trypsin (Promega, catalog number V5111) was used to generate peptides. Trypsin is a serine protease that specifically cleaves at the carboxylic terminus of lysine and arginine residues. When proline is at the carboxylic side of lysine or arginine, the peptide bond is almost completely resistant to cleavage by trypsin. Two trypsin missed cleavages were allowed. Carbamidomethyl (C) was set as a static modification. Oxidation (M), as well as acetyl (protein N-term), were selected as variable modifications. Mass accuracy was set at 4.5 ppm for precursor and 0.5 Da for the product mass tolerance. Peptides were filtered for high confidence (FDR < 1%) using a fixed value validator. Intensity-based absolute quantification (iBAQ) enabled for label-free quantification, where iBAQ values are calculated by a MaxQuant algorithm (sum of peak intensities of all peptides matching to a specific protein/number of theoretical peptides). Match between runs was enabled and set to a 1-min window. All samples were run in triplicate for three independent biological replicates. For data analysis, iBAQ values were log₂-transformed and normalized by subtracting to each value the average value of the respective sample. The peptide relative ratio was calculated using the total area under the extracted ion chromatograms of all peptides with the same amino acid sequence (including all its modified forms) as 100%. For isobaric peptides, the relative ratio of two isobaric forms was estimated by averaging the ratio for each fragment ion with a different mass between the two species. Next, extracted ion chromatography of those *m/z* ions with a mass tolerance of 10 ppm was performed. Statistical significance was assessed using a two-tail heteroscedastic *t* test (*p*-value representation ∗ =  <0.05, ∗∗ = <0.005, ∗∗∗ = <0.0005).

### Protein–Protein Interaction Model

Search tool for recurring instances of neighboring genes (STRING) v.10 database (string.org, (51)) parameters were set to include protein–protein interactions based on evidence, sourced from neighborhood, experiments, databases, co-occurrence, and coexpression. The minimum required interaction score was required to be of high confidence (0.7) with a maximum of 100 proteins interacting in the first shell and 100 proteins in the second shell. Data from peptide pulldown studies were pooled from Singh *et al.*, 2020, and Hoeijmakers *et al.*, 2019, and filtered only to include proteins that were significantly enriched in the respective studies. Finally, protein–protein interactions were derived from a yeast-two-hybrid study (52). Taken together, proteins were imported into Cytoscape (version 3.8.2). Proteins were further filtered to include only known chromatin associated proteins and proteins that have homology to known H3K18ac and H3K23ac associated proteins.

### Experimental Design and Statistical Rationale Analysis

The overall aim of this study was to identify combinations of histone PTMs and their theoretical cross talk patterns on intact histone N termini across three biologically unique stages of the *P. falciparum* parasite. For the middle-down proteomics, we analyzed two independent biological replicates for immature and mature gametocytes and three independent biological replicates for asexual trophozoite stages. The use of multiple biological replicates allowed for the assessment of variance in the data and the identification of outliers. By normalizing and averaging the relative abundances of proteins identified across biological replicates, the data are more accurately representative of actual protein abundance as compared with technical replicates. For the ChIP-MS experiments, we analyzed all samples (asexual trophozoites, immature and mature gametocytes) in three independent biological replicates. In all statistical comparisons, *p*-values were corrected for multiple testing according to Benjamini–Hochberg. Statistical analysis was performed using GraphPad Prism v6.00 and R v3.5.3, assuming normal distributions. Unless otherwise noted all error bars represent the standard error of the mean for at least three independent biological replicates.

## Results

### Adapting Middle-down Proteomics for *P. falciparum* Parasites

**Fig. 1:**
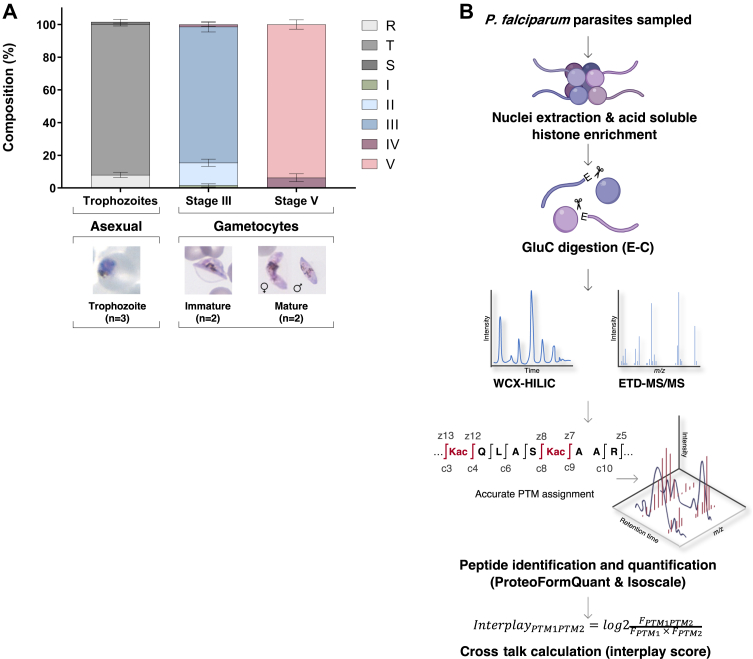
**Middle-down MS workflow for the analysis of *P. falciparum* parasite.***A*, the stage composition of the three biological stages analyzed in this study and representative morphology. Trophozoite (mean ± SEM) samples contained a small percentage of ring (R) and schizont (S) stages, whereas the stage III samples (mean ± SD) contained stage I (I), II (II), III (III), and IV (IV), and stage V samples (mean ± SD) consisted of stage III, IV, and V gametocytes. The trophozoites were from three independent biological repeats and gametocyte stages from two independent biological repeats. *B*, the middle-down proteomics workflow. Histones were enriched from trophozoite, stage III, and stage V gametocytes and digested using endoprotease GluC, which cleaves at the C terminus of glutamic acid residues, generating intact N-terminal histone H3 peptides (50 amino acid residues in length). To allow sample loading in aqueous buffer and for the most efficient separation for histone N-terminal tails, nano liquid chromatography equipped with a two-column system consisting of a C18-AQ trap column and a weak cation exchange-hydrophilic interaction chromatography (WCX-HILIC) resin analytical column coupled online with high-resolution tandem mass spectrometry (MS-MS) fragmentation was performed using electron transfer dissociation (ETD). Spectra were identified using the Mascot and peptides were quantified using isoScale. The flask image was adapted from the image from Servier Medical Art (http://smart.servier.com/). Servier Medical Art is licensed under a Creative Commons Attribution 3.0 License (CC BY 3.0 license: https://creativecommons.org/licenses/by/3.0/). Data processing and analysis workflow involved MS spectral deconvolution using Xtract (Thermo Fisher Scientific) followed by database searching using Mascot (Matrix Science) with files generated from PlasmoDB (https://plasmodb.org/) and subsequent removal of ambiguously mapped PTMs and stringent quantification (including cofragmented isobaric species) using IsoScale Slim (http://middle-down.github.io/Software). The relative abundance of an individual PTM (PTM1/2) is calculated by summing the relative abundances of all proteoforms carrying the specific individual PTM. The interplay between two individual modifications is calculated by dividing the observed abundance of bivalent PTMs (FPTM1PTM2) with the predicted frequency of a combinatorial PTM (FPTM1 x FPTM2). FPTM1PTM2 is calculated by summing the relative abundances of all proteoforms carrying both PTMs. PTM, posttranslational modification.

To investigate the presence of coexisting histone PTMs with middle-down proteomics, we isolated parasites as enriched trophozoites (92 ± 0.9%), immature early-stage gametocytes (83 ± 3.1% stage III gametocytes, 14 ± 2.2% stage II), and mature gametocytes (94 ± 2.9% stage V), to allow stage-specific differences to be inferred (Fig. 1*A*). All samples yielded >300,000 cells/sample, resulting in sufficient yields of acid-soluble nuclear protein fractions containing the histones: 17 ± 1.1 μg for the trophozoite population and 58 ± 10 μg and 70 ± 16 μg for the immature and mature gametocytes, respectively (supplemental Fig. S1*A*).

An optimized middle-down proteomics workflow was established to accurately identify and quantify individual and combinatorial histone PTMs at scale (Fig. 1*B*) (45, 53). The native histones from each sample were digested with GluC endoproteinase to produce polypeptide fragments of ∼50 to 60 amino acids in length (≥5 kDa) that were separated and identified by high-resolution nano liquid chromatography-MS/MS; data processing and peak extraction were performed with in-house developed tools, ProteoFormQuant and HistoneCoder (48, 54). Unique PTMs (with an FDR <1%) with sufficient fragment ions (supplemental Fig. S1*B*) were accurately quantified, with a coefficient of variance ≤33% and average Pearson r^2^ = 0.69 between biological replicates (supplemental Fig. S1*C*), allowing comparison of PTM quantities between samples. To ensure differentiation of isobaric peptides as a result of the middle-down proteomics, data-dependent acquisition data were further processed using isoScale Slim (37), which extracts the total fragment ion intensity of histone tail spectra as representative of their abundance, and it discriminates isobaric forms using unique site-specific fragment ions using the principle of the fragment ion relative ratio (55). From the accurately identified and quantified histone PTMs per peptide, the coexisting frequency was calculated for the observed presence of combinatorial PTMs (from MS2 level evidence) as a function of predicted coexistence frequencies, referred to as an “interplay score” (55, 56, 57).

### A High-resolution, Quantitative Compendium of Histone PTMs in *Plasmodium* From Middle-down Proteomics

**Fig. 2:**
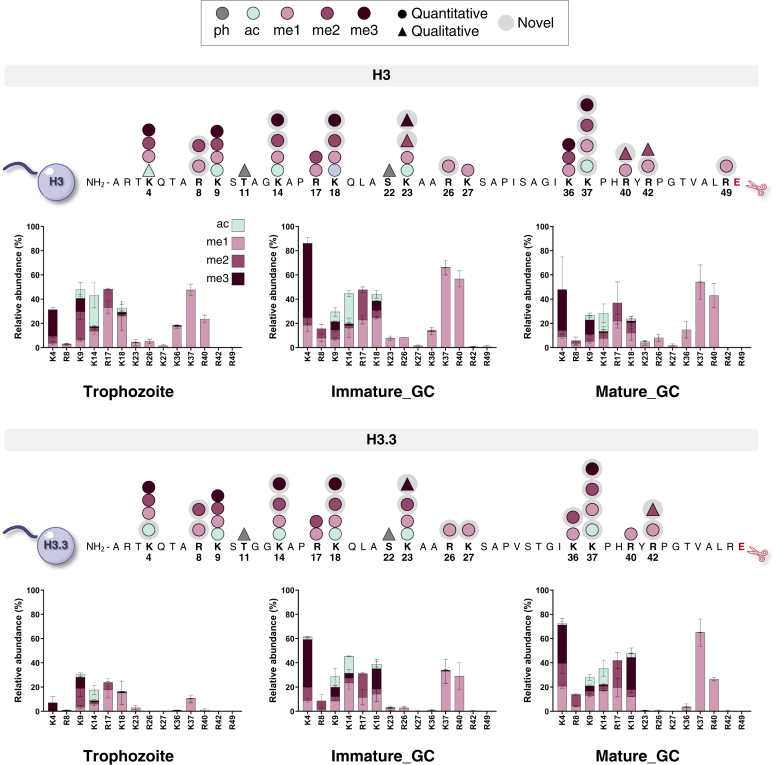
**The relative abundances of individual PTMs on histone H3 and variant histone H3.3 from *P. falciparum* trophozoite and immature and mature gametocytes.** All individual histone PTMs that were detected for histone H3 and H3.3 are indicated. A total of 77 PTMs on histone H3 and variant histone H3.3 were identified across all stages, including 66 quantitative (*circle*) and 11 qualitative histone PTMs (*triangle*) of which 34 were novel and detected for the first time in *P. falciparum* parasites (*gray shaded*). Abbreviations denote the PTMs that included mainly acetylation (ac), mono-, di-, and trimethylation (me1, me2, and me3, respectively), and phosphorylation (ph). The N-terminal peptide fragmented for analysis is shown with the corresponding amino acid sequence. The relative abundances of the individual histone PTMs on histone H3 and histone variant H3.3, showing relative abundances on the different histone positions with acetylation (*teal*), monomethylation (me1, *light purple*), dimethylation (me2, *medium purple*) and trimethylation (me3, *dark purple*). Data are from three (for trophozoites) or two (immature and mature gametocytes) independent biological repeats, mean ± SEM. PTM, posttranslational modification.

The high-resolution, comprehensive nature of middle-down proteomics allowed successful identification and quantification of 77 PTMs on histone H3 and H3.3 (Fig. 2), as only these histones have GluC cut sites on their N-terminal histone tail (45). Histone H2B.Z PTMs were also identified but were not further analyzed owing to incomplete cleavage concerns. Mass tolerance was set at 30 ppm to accurately distinguish between lysine acetylation (42.011 Da) and lysine trimethylation (42.047 Da) as previously determined (49). Of the 77 identified PTMs, 66 were confidently quantified; the others were only detected but with a signal insufficient to assess an abundance (Fig. 2). As no enrichment was performed for phosphorylation, these could not be quantified. A few PTMs previously identified with H3 (S10ac, S10ph, S28ph, S32ph) and H3.3 (S10ac, T11ac, S22ac, S28ph, S32ph) could not be confirmed (22, 29). Since these PTMs were only previously qualitatively described, their presence remains to be confirmed.

Of the 77 PTMs identified (Fig. 2), 34 were described for the first time in *P. falciparum* parasites and 66 could be accurately quantified. Histone H3 contained 40 PTMs of which 33 were accurately quantified, including 16 novel PTMs. These include acetylation and methylation of H3K37 and methylation of several arginines (H3R8me1/2, H3R26me1, H3R40me2, H3R42me1, and H3R49me1). Several PTMs displayed high relative abundances (>20%) in more than two stages (*e.g.*, H3K4me3, H3K14ac, H3R17me1, H3K18me1, H3K37me1, and H3R40me1), with the novel PTM, H3K37me1, highly abundant in immature gametocytes (70 ± 6%) (Fig. 2). These changes in abundance levels between stages are also evident for H3K4me3, H3R17me2, and H3R40me1, which increased significantly from trophozoites to immature gametocytes (*p* ≤ 0.01, n ≥ 2, supplemental Fig. S2). Histone H3.3 contained 37 PTMs (33 quantified) with substantially higher abundances of PTMs modified in both gametocyte stages compared with asexual parasites than seen for H3. Methylation PTMs were again abundant, including H3.3K4me1/3, H3.3K18me3, H3.3R17me2, and H3.3R40me1, with H3.3K37me3 with significantly increased abundance in mature gametocytes (*p* ≤ 0.01, n = 2, supplemental Fig. S2).

Collectively, we demonstrate that middle-down MS identified >70 unique PTMs and quantified 86% thereof. This includes 34 novel modifications, thereby providing an updated, high-resolution compendium of histone modifications across multiple life cycle stages of *P. falciparum*.

### A Stage-specific Combinatorial Histone PTM Code Exists in *P. falciparum* Parasites

**Fig. 3:**
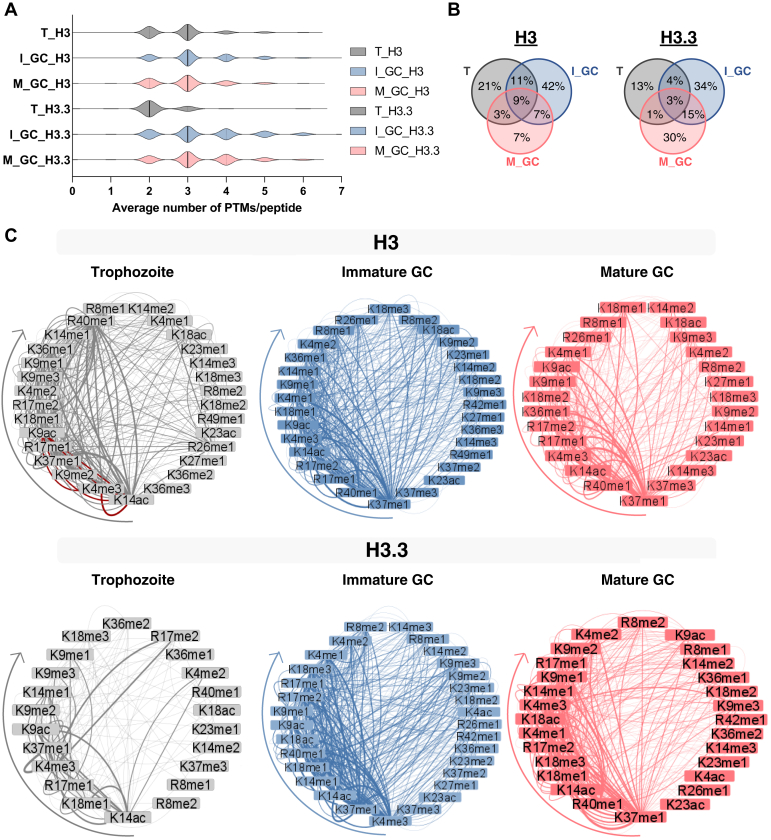
**Prominent combinatorial histone PTM reorganization in *P. falciparum* parasites and gametocytes.***A*, violin plots show the distribution of the number of PTMs that are present on histone H3 and variant H3 tails where the *dotted line* indicates the upper and lower quartiles and the *solid line* indicates the median. The lower limit represents an unmodified histone tail. T, trophozoite. *B*, the Venn diagram indicates that combinatorial peptides shared between the three stages from histone H3 and H3.3 were relatively unique to each stage [trophozoites, *gray*; immature gametocytes (I-GC; stage III), *blue*; mature gametocytes (M_GC; stage V), *pink*] sharing only 9% and 3% of combinatorial peptides, respectively. *C*, the coexisting histone PTMs observed on histone H3 and H3.3 for trophozoites, I-GC (stage III), and M_GC (stage V) are visualized as ring plots where the nodes are the histone PTMs and the edges represent the connection to another coexisting partner PTM. All combinations are included in the supplemental data. The *arrows* represent the start of the most connected PTM (*left*) toward the least connected (*right*) in a clockwise direction. The coexistence of H3K9ac with H3K4me3, H3K14ac with H3K4me3, and H3K9ac with H3K14ac is highlighted with *red edges* in the trophozoite stage. The *arrows* indicate the most prevalent PTM to the least prevalent PTM in coexistence. PTM, posttranslational modification.

The middle-down proteomics dataset was used to identify and quantify coexisting PTMs on H3 and H3.3 across the three life cycle stages of *P. falciparum*. On average, three coexisting PTMs were present on any given peptide for both H3 and H3.3 (Fig. 3*A*), like what is seen for these two histones in humans and mice (33, 58, 59). H3.3 is the exception, with on average only two coexisting PTMs present in trophozoites but as many as seven (*e.g.*, H3K4me1R8me1K9me1K14acR17me1K18acK37me1) in immature gametocytes (Appendix 1).

Stage stratification was evident in the histone PTM combinations, with immature gametocytes displaying the highest proportion of unique combinations (42%) on H3, with only a minor proportion (9%) of combinations shared between asexual parasites and immature and mature gametocytes (Fig. 3*B*). Stage-specific diversity was somewhat less evident for H3.3 (Fig. 3*B*), with a markedly decreased number of coexisting PTMs present in trophozoites for this histone (Fig. 3*C*). The stage stratification was characterized by changes in the identity of the most prevalent combinations. Trophozoites are characterized by a large number of combinations involving H3K14ac, H3K4me3, H3K9me2, and novel PTMs H3K37me1 and H3R17me1 (supplemental Fig. S3). These include the well-characterized, known combination of the archetypical euchromatic PTMs H3K4me3 and H3K9ac (21, 60), with H3K14ac due to the coordinated action of SET7 (26) and the histone acetyltransferase general control nonrepressed 5 protein acetyltransferase (GCN5) (61, 62). In gametocytes, the novel PTMs H3K37me1 and H3R40me1 are involved in the highest number of coexisting PTMs, with further specification seen between immature gametocytes (with frequent interactions with H3R17me1&2 present) and mature gametocytes (higher connectivity for H3K14ac and H3K4me3 than H3R17me1). Combinations involving H3R42me1 occurred exclusively in gametocytes. Arginine methylation may therefore well make up a key component of the histone code in *P. falciparum* parasites along with other histone PTMs, particularly for gametocyte stages.

These data indicate that histones are rarely modified by single, individual PTMs. Therefore, multiple histone PTMs, as opposed to a single PTM on a particular histone tail, would contribute to a distinct chromatin structure that could promote gene expression. As well, these histone codes are rearranged in position and type of modifications in different life cycle stages of the malaria parasite. The most frequent combinations in gametocytes diverge from those trophozoites, with immature gametocytes associated with the highest number of coexisting PTMs ascribed to the presence of novel PTMs. The parasite, therefore, employs a unique and diverse set of PTM combinations likely to guide stage-specific gene expression.

### Histone PTM Pairs Display Unique Cross Talk to Assert Function

The extent and relevance of the influence between pairs of coexisting PTMs were subsequently interrogated by quantifying the coexisting frequency as an interplay score (IS) for bivalent combinations (Appendix 2). This provides a metric to predict the likelihood of individual pairs of histone PTMs to either (1) occur more frequently as coexisting pairs than expected by chance (49, 56, 63) or (2) have a negative interplay score to indicate that two individual pairs of PTMs are observed less frequently than expected by chance. Thus, if two PTMs have a positive interplay score, it indicates that these PTMs likely enhance or disrupt the binding of effector proteins and, therefore, share a “codependent” relationship to one another. If a negative interplay score is observed, it means that the PTMs act in an antagonistic or mutually exclusive manner from one another, indicating functional independence (64). If two PTMs are randomly and independently deposited on chromatin, they will have an interplay score close to zero.

**Fig. 4:**
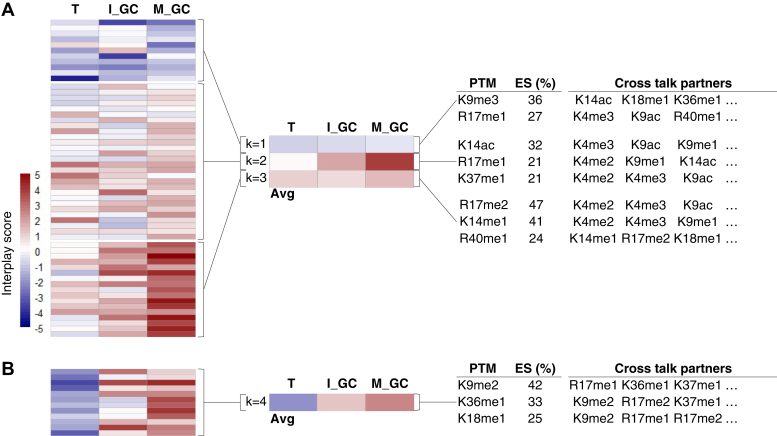
**Histone modification cross talk conserved across life cycle stages in *P. falciparum*.***A*, interplay scores for PTM combinations are consistently present in all life cycle stages analyzed. Heatmaps show k-means clustering (complete, k = 4) of the overlapping 35% or 68 bivalent combinations and the respective interplay scores shared between all three stages. The average interplay scores are summarized for each cluster. *B*, interplay scores in cluster 4 across the life cycle stages and PTMs that show enrichment within these clusters are quantified with an enrichment score. An enrichment score percentage (ES %) is shown for the top PTMs that are overrepresented in each cluster. The enrichment score is calculated by dividing the total number of times a given PTM is present by the total number of combinations in the cluster. Selected cross talk partner PTMs are indicted, with all the combinations provided in Fig. S4. PTM, posttranslational modification.

A third of all coexisting histone PTMs had similar connectivity and coexisting frequency profiles between all three stages of development of *P. falciparum* (Fig. 4 and supplemental Fig. S4, *A* and *B*). Of these, 11 combinations show negative interplay scores throughout development (Fig. 4*A*), which includes multiple combinations of the repressive PTM H3K9me3 (supplemental Fig. S4*C*). For example, this PTM is found on the same histone molecule with H3K14ac significantly more rarely than stochastic co-occurrence in all three stages, implying mutual exclusion and thus opposing biological functions. The typically euchromatic signal of H3K14ac is therefore negated in the event of H3K9me3, and this contributes to the HP1-bound heterochromatic state as described for certain gene sets (23, 65, 66). This combination is also found in differentiated stem cells (67) where H3K9me3 acts as a barrier to cell reprogramming induced in pluripotent stem cells, regardless of the presence of the activating H3K14ac PTM (68). All other combinations with H3K9me3 have negative interplay scores (supplemental Fig. S4). H3K9me3, therefore, likely act autonomously, independent of association with any other acetylation or methyl PTMs, and not influenced by *P. falciparum* life cycle development. This feature of H3K9me3 is supported in other cell types where H3K9me3 acts to reprogram the identity of various cell types (69, 70).

Several PTMs (45) display positive interplay throughout development, implying coordinated function or codependence including H3R17me2, H3K14me1, and H3R40me1 found in various combinations with partner PTMs, particularly H3K4me2/3 (Fig. 4*A* and supplemental Fig. S4). These marks therefore likely never function on their own and need interaction with one (or more) PTMs throughout parasite development. Of interest, a number of these marks show increased positive interplay scores in mature gametocytes, suggesting that these combinations are increasingly critical to these parasites. H3K14ac remains codependent on H3K9ac as seen before for asexual parasites (61, 62) with pronounced dependency evident in gametocytes here (Fig. 4); indeed, the effector protein GCN5 is expressed in all three stages investigated, including mature gametocytes (71).

A set of 12 combinations are found in all three life cycle stages, yet have opposing profiles between gametocytes (strong positive interplay scores) and asexual parasites (strong negative interplay) (Fig. 4*B* and supplemental Fig. S4). This includes several combinations involving H3K9me2, H3K36me1, and H3K18me1. The repressive mark H3K9me2 associates with several other marks (*e.g.*, R17me1, K18me1, K36me1, K37me1, and R40me1), all of which show codependency only in mature gametocytes. The strongest differential interplay score between asexual parasites and gametocytes was observed for H3K4me3K23me1. This novel combination is mutually exclusive in trophozoites, supporting the fact that H3K4me3 participates in cross talk with H3K9ac in asexual parasites (21). The strong codependence of H3K4me3K23me1 is therefore likely important for gametocyte-specific biological processes; the combination of the euchromatic H3K4me3 with H3K23me1 methylation has been associated with heterochromatin in *Caenorhabditis elegans* (72).

The conserved nature of these coexisting marks across all life cycle stages of *P. falciparum* implies shared importance to parasite biology.

### A Dynamic Histone Code Describes Cross Talk With Stage-specific Biology in *P. falciparum*

**Fig. 5:**
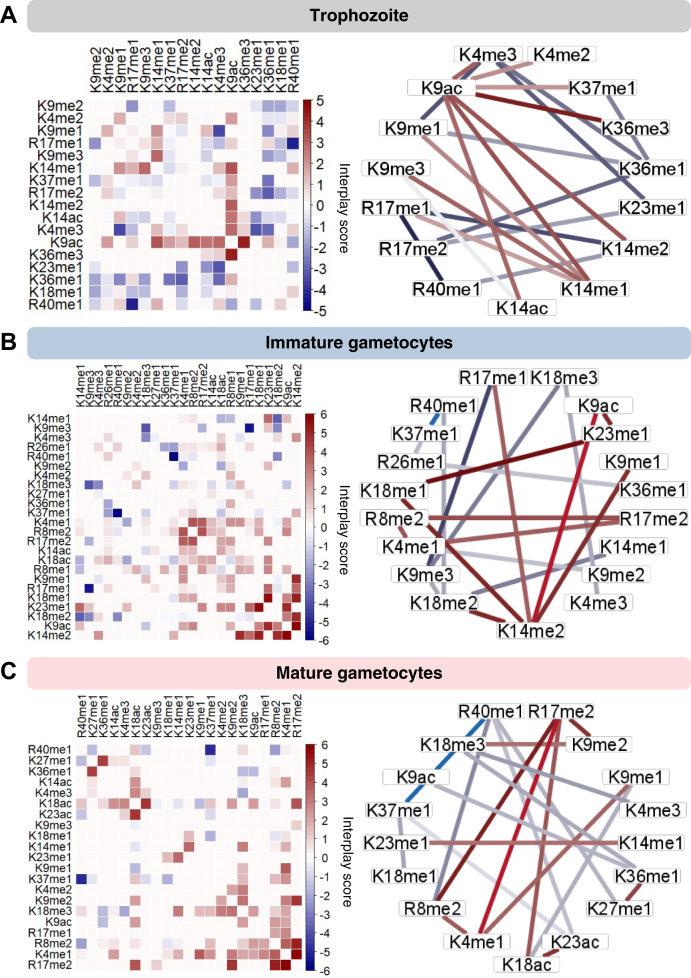
**Dynamic, stage-stratified histone modification cross talk in *P. falciparum* parasites.** Interplay scores between bivalent posttranslational modifications occurring in (*A*) trophozoites, (*B*) immature gametocytes, and (*C*) mature stage gametocytes where the color intensity of the square is proportional to the interplay score. Ring plots indicate top and bottom 10 bivalent combinations, where the color of the edges is proportional to the interplay score shown in the heatmap. Interplay scores for trophozoites include the unique 4% and the overlapping 6% with immature gametocytes (I_GC) and 1% with mature gametocytes (M_GC). Interplay scores for immature gametocytes include the unique 25% and the overlapping 6% with trophozoites and 25% with mature gametocytes. Interplay scores for mature gametocytes include the unique 6% and the overlapping 4% with trophozoites and 25% with immature gametocytes.

Several PTMs show a cross-talk profile that is uniquely associated with a specific parasite life cycle stage (Fig. 5 and supplemental Fig. S5). In trophozoites, the majority of the PTM combinations had negative interplay scores, suggesting that these PTMs antagonize each other and are thus mutually exclusive. Combinations including novel arginine methylation marks like H3K14me2R17me1 show the strongest negative interplay scores (IS = −3.7), suggesting that these marks act with different biological roles from each other in trophozoites (Fig. 5*A*). Both H3R40me1 and H3R17me1 mostly show pronounced negative interplay scores for most of their interactions in trophozoites. H3R17me1 activates as a derepressor in mammalian cells (73), whereas H3R40me1 has only previously been reported in a few organisms including yeast where it is required for efficient sporulation, similar to spermatogenesis in higher eukaryotes (74, 75), whereas in cancer cells (76), *C. elegans* (34), and murine embryonic stem cells, its function is unknown (49).

Several important codependent interactions with positive interplay scores were identified in trophozoites, the majority of which involved H3K9ac (*e.g.*, H3K9acK14ac IS = 2.8; H3K4me3K9ac IS= 2.9; and H3K9acK36me3) to produce the global euchromatic signal associated with asexual parasites (20, 21). H3K9acK36me3 has the strongest positive interplay score (IS = 4.3), and both H3K9ac and H3K36me3 have been independently linked to *var* gene transcription (20, 25, 77, 78, 79); this codependence supports their function in *var* gene expression.

The cross-talk profile in immature gametocytes is more complex, with a larger number of PTMs involved in positive cross talk, the majority of which involve methylation marks (Fig. 5*B*). The novel mark H3K14me2 displays the largest number of interactions with positive cross talk, including that with H3K9ac (IS = 6), H3K9me1 (IS = 4.9), and both K18me1&2 (IS ∼ 5). These associations are influenced by the level of methylation (*i.e.*, mono-, di-, or trimethylation) to define chromatin states and active genes *versus* inactive genes within the same locus (80, 81, 82), with H3K14me1, for instance, in negative cross talk with H3K18me2, compared with the positive cross talk seen for H3K14me2 with H3K18me2. H3K14me2 is likely a repressive mark, like the important silencing function of H3K14me3 that marks a set of zinc finger protein genes during transdifferentiation of bone marrow cells into hepatocytes (83, 84). The codependency with H3K14me2K9me1, with H3K9me1 as key PTM in the establishment of functional heterochromatin (85) and the novel mark H3K14me2K18me1/2, marks H3K14me2 as a key modulator of gene regulation within immature gametocytes, to potentially mediate the establishment of a heterochromatic state.

Among the novel arginine PTMs in immature gametocytes, H3R17me2 shows positive interplay particularly with H3R8me2 (IS = 4.1, correlated only with active histone PTMs (86)) and H3K4me1 (IS = 3.6, enhancer associated activation mark (87)), with the latter also positively connected (H3K4me1R8me2, IS = 3.97). H3R17me2 is a typical activation mark in eukaryotes (88, 89, 90), and the coordination with the other two activation marks indicates that these combinations may be involved in specific activation processes. Interesting, changing H3R17 methylation from di- to monomethylation results in not only positive interactions with H3K14me2 (IS = 3.59) but also a strong negative interaction with H3K9me3 (IS = −5.33), implying independence of H3R17me1 from H3K9me3. Furthermore, in immature gametocytes, H3R40me1 again shows strong negative interplays, particularly with H3K37me1 (IS = −7) and H3K18me2 (IS= −2.8), confirming its independent action in immatures gametocytes, like in trophozoites. Overall, immature gametocytes use highly connected and codependent repressive lysine PTM pairs to induce a more heterochromatic state compared with asexual parasites. However, most of the arginine PTMs coordinate positively and could result in the activation of subsets of genes.

Mature gametocytes have a combinatorial profile similar to that of immature gametocytes with largely positive interplays scores (Fig. 5*C*), although the frequency of the combinations somewhat differs from those in immature gametocytes. H3R40me1 remains highly connected in mature gametocytes, again showing independence from other coexisting marks. Activating H3R17me2 is the most connected PTM in mature gametocytes, retaining its interactions and codependency with other activating marks, H3K18ac (IS = 3.9) and H3K4me1 (IS = 5.9), and with H3K9me2 (IS = 4.8) and H3R8me2 (IS = 5.2). Mature gametocytes are, however, additionally marked by combinations that uniquely and exclusively are present only in this stage of development. This includes codependency between H3K27me1K36me1 (IS = 4.3). In embryonic stem cells, H3K27me1 is dependent on H3K36me1 to promote transcription (54) and an increase in H3K36 methylation to di- and trimethylation negatively impacts H3K27 function and they become mutually exclusive (91, 92). A likely explanation for this positive interplay between H3K27 and H3K36 is that, when both sister histones of mononucleosomes carry H3K36, methylation of H3K27 by the polycomb repressive complex 2 is inhibited (93). In *P. falciparum*, H3K36me2/me3 is repressive to asexual gene sets in immature gametocytes (25). The exclusive combination of H3K27me1K36me1 in mature gametocytes could therefore be predicative of transcriptional activation of genes required for parasite transmission. In addition, H3K18ac also is in cross talk with H3K23ac only in mature gametocytes (IS = 4.7 for H3K18acK23ac). H3K18ac independently associates with active promotors in asexual *P. falciparum* parasites (94) and human cancer cells; deacetylation of H3K18ac by sirtuin protein 7 results in transcriptional repression (95, 96). Furthermore, the codependency between H3K18ac, H3K23ac, and H3R17me2 has been demonstrated to induce transcriptional activation in cancer cells (97) and could therefore similarly be critical for stage-specific gene expression exclusively in mature gametocytes. This coordination may be an essential requirement for subsequent gamete formation and fertilization. The histone code in mature gametocytes may therefore result in bistable chromatin to enable a transcriptionally poised state of some genes for rapid fertilization and sexual replication once these mature gametocytes are taken up by a feeding mosquito, and this is mediated by unique codependent histone PTM combinations.

### Shared Protein Effectors Coordinate to Mediate H3K18acK23ac Function in Mature Gametocytes

**Fig. 6:**
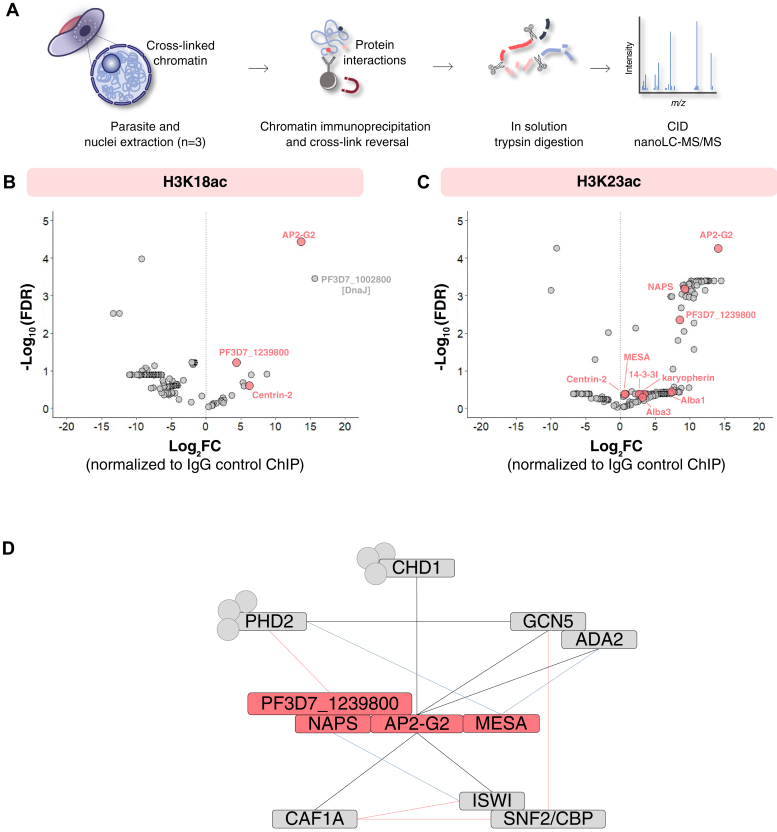
**Proteins identified by chromatin proteomic profiling are associated with H3K18ac and H3K23ac in mature stage gametocytes.***A*, after parasite DNA and proteins were cross-linked, nuclei were isolated and chromatin was sonicated. Chromatin complexes were immunoprecipitated with antibodies raised against histone posttranslational modifications H3K18ac and H3K23ac. Cross-links were reversed, and proteins trypsin digested. NanoLC coupled with collision-induced dissociation (CID) tandem mass spectrometry (MS/MS) was performed, followed by database searching using Thermo Proteome Discoverer (v.1.4.1.14) to extract peaks, Scaffold (v.4.5.3) to validate and quantify peptides, Mascot (v.2.5.1) to identify proteins, and proteins identified searches using databases PlasmoDB (v.46) and UniProt (2020_01). Proteins were quantified using intensity-based absolute quantification values. Images were adapted from Servier Medical Art (URL link to the license: https://creativecommons.org/licenses/by/3.0/), and changes were made in terms of color, size, and composition. Scatter plot of the (*B*) H3K18ac- and (*C*) H3K23ac-associated proteome enrichment in the Chromatin immunoprecipitation (ChIP)–mass spectrometry. A total number of 282 proteins were identified in the preparations, with several proteins showing positive log₂-fold change normalized to the negative control IgG ChIP. Proteins that are shared between the H3K18ac and H3K23ac samples include transcription factor AP2-G2 (PF3D7_1408200), a conserved unknown function protein (PF3D7_1239800) and centrin-2 (PF3D7_1446600) as shown in pink. H3K23ac also includes nucleosome assembly protein (PF3D7_0919000, NAPS), DNA/RNA-binding protein Alba 1 (PF3D7_0814200, Alba1), karyopherin beta (PF3D7_0524000), DNA/RNA-binding protein Alba 3 (PF3D7_1006200), 14-3-3 protein (PF3D7_0818200, 14-3-3I), and mature parasite-infected erythrocyte surface antigen (PF3D7_0500800, MESA). *D*, a schematic model of the protein–protein interaction complex manually curated and placed in a network based on evidence identified in this study, an AP2-G2 peptide pulldown study (101) and a GCN5 pulldown (103), previous yeast two-hybrid protein interactions (52) and STRING interactions. Shown in *gray blocks* are interactions that include the transcriptional coactivator ADA2 (PF3D7_1014600, ADA2), histone acetyltransferase GCN5 (PF3D7_0823300, GCN5), chromodomain-helicase-DNA-binding protein 1 homolog (PF3D7_1023900, CHD1), ISWI chromatin-remodeling complex ATPase (PF3D7_0624600, ISWI), Snf2-related CBP activator (PF3D7_0820000, SNF/CBP), chromatin assembly factor 1 subunit A (PF3D7_0501800, CAF1A), and PHD finger protein PHD2 (PF3D7_1433400, PHD2). *Gray dots* represent other proteins that associate with the respective proteins but are inconsequential. Proteins shown in *pink blocks* are shared between the H3K18ac and H3K23ac samples and include transcription factor AP2-G2 (PF3D7_1408200), nucleosome assembly protein (PF3D7_0919000, NAPS), a conserved unknown function protein (PF3D7_1239800) and mature parasite-infected erythrocyte surface antigen (PF3D7_0500800, MESA). *Black edges* represent data from the AP2-G2 and GCN5 interactomes (101, 103); the *pink line* represents data from STRING (STRING: functional protein association networks [string-db.org]); and the *blue line* indicates data from yeast to hybrid study (52).

The unique combination of H3K18acK23ac, and the associated coexistence with H3R17me2 in mature gametocytes, warranted further investigation of the presence of shared effector proteins to promote a biological outcome of transcriptional activation associated with these combinations. To identify the protein machinery associate with these marks, we performed quantitative chromatin immunoprecipitation on cross-linked chromatin isolated from mature gametocytes, coupled with quantitative mass spectrometry (ChIP-MS, Fig. 6*A*). The presence of both marks found in combination was validated by Western blot analysis in mature gametocytes (supplemental Fig. S5*A*). Since an antibody against H3K18acK23ac is not commercially available, we opted to capture proteins that interact with or are recruited to both H3K18ac and H3K23ac, respectively, in two separate ChIP experiments using antibodies specific to H3K18ac and H3K23ac (supplemental Fig. S5B). We used this technique to ensure that any endogenous protein (or protein complexes) in mature gametocytes that associate with these marks are captured in its *in vivo* context (98).

We defined proteins significantly associated with H3K18ac and H3K23ac as those with a log_2_ fold change ≥2 for the ChIP population (cross-linked chromatin preparation) compared with the IgG control ChIP, at an FDR ≤5%. Selective enrichment of proteins for H3K18ac and H3K23ac was confirmed by (1) the significant (*p* ≤ 0.01) enrichment of unique peptides for low-abundance proteins in the H3K18ac and H3K23ac ChIP preparations compared with the IgG sample preparations (supplemental Fig. S5*C* and Appendix 4); (2) significantly increased abundance of proteins in the histone PTM samples compared with the IgG samples based on relative intensity-based absolute quantification (iBAQ) (supplemental Fig. S5*D*, *p* ≤ 0.01); and last, (3) the enrichment in the ChIP population for chromatin associated proteins (>20% enrichment) (99), the nuclear pore proteome (100), and transcription factor interacting proteins (101) (supplemental Fig. S5*E*).

We found three proteins to be strongly enriched for H3K18ac in the ChIP-MS data, compared with 46 proteins for H3K23ac (FDR <5%, log_2_ fold change ≥2 over IgG control ChIP) (Fig. 6). The transcription factor AP2-G2 (PF3D7_1408200), a protein with an unknown biological function (PF3D7_1239800), centrin-2 (PF3D7_1446600), and DnaJ (PF3D7_1002800) were enriched in both PTM ChIPs. Besides the chaperone DnaJ, all these proteins were previously implicated to be chromatin associated (99). The gene for PF3D7_1239800 is refractory to deletion, is likely essential for asexual parasite survival (30), and is highly expressed in mature gametocytes (71). AP2-G2 is also essential for the successful completion of gametocyte maturation and transmission (101, 102).

To further investigate the epigenetic complexes associated with H3K18ac and H3K23ac, protein-protein interaction data were used to assemble a proposed reader complex for these PTMs in mature stage gametocytes, associated with AP2-G2 (Fig. 6*C*) (52, 101, 103) (Appendix 3). Transcription factors are indeed observed in many histone PTM-associated complexes (103) to recruit additional members of epigenetic complexes, as shown for AP2-I (104). The complex included evidence for the direct interaction between GCN5 (PF3D7_0823300), with acetyltransferase activity, and ADA2 (PF3D7_1014600), pointing toward the involvement of a histone acetyltransferase Spt-Ada-Gcn5 acetyltransferase (SAGA)-like complex. This was supported by AP2-G2 further interacting with the chromodomain-helicase-DNA-binding protein 1 homolog (PF3D7_1023900; CHD1), which in turn interacts with plant homeodomain (PHD) 2 (PF3D7_1008100), a parasite-specific PHD-finger domain-containing protein with different specificity to its PHD1 partner (unable to bind, *e.g.*, H3K4me3 (103)). The nucleosome assembly protein (NAPS) and PF3D7_1239800 also have direct interactions with PHD2. In gametocytes, H3K18acK23ac, therefore, associates with a GCN5-ADA2-PHD2 SAGA-like complex *via* the tight interaction of AP2-G2, NAPS, and PF3D7_1239800 as binding partners to this histone combination. Most of these proteins are predominantly expressed in male mature gametocytes compared with female gametocytes, including ADA2, PHD2, ISWI, NAPS, CHD1, and AP2-G2 (105), implicating these proteins in downstream sex-specific chromatin structure changes.

AP2-G2 additionally interacts with other chromatin regulation proteins, including a chromatin assembly factor 1 subunit A (PF3D7_0501800, CAF1A) and an ISWI chromatin-remodeling complex ATPase (PF3D7_0624600, ISWI), which interacts with a sucrose nonfermentable (Snf2)-related CREB (cAMP response element-binding protein)-binding protein (CBP) activator (PF3D7_0820000). As a result, AP2-G2, H3K18ac, H3K23ac, ISWI chromatin-remodeling complex ATPase (PF3D7_0624600, ISWI), and Snf2-related CBP activator (PF3D7_0820000) share a link. AP2-G2 is associated with H3K18ac and H3K23ac and interacts with ISWI, which has a relatively high homology to TRIM24 (42% identity), which is a reader of H3K18ac and H3K23ac (106, 107). Furthermore, the writers of H3K18ac and H3K23ac, p300/CBP activator (108, 109, 110), have homology to the *P. falciparum* histone acetyltransferase, GCN5 (PF3D7_0823300, 34% identity). Furthermore, TRIM24 has been described as CBP-associated proteins (111). This suggests that the SAGA-like complex of *P. falciparum* has as core GCN5/ADA2/PHD2 but in gametocytes, association with the H3K18acK23ac combination includes the additional ISWI/SNF complex effectors. This cooperation between SAGA and SWI/SNF complexes is required to regulate specific transcriptional responses, as in yeast (112). Since SWI/SNF complex proteins are global nucleosomal organizers that enable the specific binding of selective transcription factors (113, 114, 115), their involvement could explain the recruitment of AP2-G2.

The involvement of Snf2/CBP further points to functionality for the cross talk involving the neighboring H3R17 mark, forming the H3R17me2K18acK23ac coordinating code in *P. falciparum* mature gametocytes. Arginine 17 methylation is achieved systematically: CBP first acetylates H3K18, then H3K23, and this allows the arginine methylase coactivator-associated arginine methyltransferase 1 (CARM1) to associate with chromatin to methylate H3R17 (116, 117). H3R17me2 is associated with transcriptional activation based on the recruitment of polymerase-associated factor 1 complex to initiate transcription in humans (89, 118), supported by the antiproliferative effects of a specific CARM1 inhibitor on multiple myeloma cell lines (119, 120). This suggests that, in *P. falciparum*, this combination could be critical for mature stage gametocytes.

Given the shared core proteins between H3K18ac and H3K23ac and the positive cross talk observed for this combination (and including R17me2), our data for this combination provide evidence that the combinatorial histone code of the *P. falciparum* parasite can recruit protein complexes unique to a combination to facilitate downstream biological processes.

## Discussion

Here we present the first systems-level evidence of a comprehensive combinatorial histone code for various life cycle stages of the human malaria parasite, *P. falciparum*. Middle-down proteomics provided high-resolution quantitative data to describe the histone code in this parasite, which could function as a model for other protista. Our data reveal that the combinatorial histone PTM landscape is dynamic, with clear stage-specific differentiation observed, with gametocyte stages more dependent on histone PTM cross talk than asexual parasites.

Collectively, our study shed light on the difference between the asexual replicating parasite and the differentiated nonreplicative gametocyte. The histone code of *P. falciparum* asexual parasites resembles that of lower eukaryotes, which has a simple genome organization and fewer histone PTMs. Given the primal function it performs, elaborate epigenetic gene regulation mechanisms may not be as important to these stages, as most of the genome is in a euchromatic state and actively transcribed during proliferation. Therefore, asexual parasites use less, and mutually exclusive, histone PTMs to regulate key functions such as host immune evasion. However, gametocytes share a similar mechanism of epigenetic gene regulation with other higher-order, multicellular eukaryotes where chromatin is predominantly condensed and highly regulated to specify the identity and purpose of a cell through multiple histone PTMs. General positive cross talk between histone PTMs is seen in gametocytes and, given the limited number of transcription factors in *P. falciparum* parasites, gametocytes could rather switch to a more complex epigenetic code to impress very specific regulation of its biological processes. This would suggest that, at least in some stages of the parasite, epigenetic level gene control is superior to transcriptional level control.

The connectivity within the histone code of *P. falciparum* is characterized by the presence of novel marks, with several new arginine modifications identified. The advances in proteomics technologies such as middle-down proteomics allow such robust description of arginine methylation marks (76) as observed here, and this contributes to our understanding of the conserved nature of arginine methylation and its key importance to chromatin organization throughout eukaryotes (88). We show that histone arginine methylation is equally as prevalent and abundant as lysine PTMs in *P. falciparum* and these marks participate in complex cross talk with one another, particularly in gametocytes. The presence of typically activating marks such as mono- and demethylation of H3R17 (88) and their codependence on other marks, *e.g.*, H3R8me2 raise interesting questions as to the importance of cooperation in activation of gene sets in gametocytes. This is extended to additional arginine methylation marks including the exclusive nature of H3R42me1 in gametocytes and the highly connected but independently functioning H3R40me1. It is noteworthy that H3R40me1 is required as activating mark for spermatogenesis-like processes in yeast (74, 75). These marks could be similarly important for activation of male gamete gene sets, supporting the notion of transcriptionally active “poised” states in mature gametocytes (24) to enable onward transmission. The importance of arginine methylation in histone PTM combinations to mediate a specific transcriptional outcome in gametocytes is therefore of interest. The parasite genome does contain the necessary machinery for arginine methylation including two putative protein arginine methyltransferases (PRMT1 [PF3D7_1426200] and PRMT5 [PF3D7_1361000]) and a putative histone arginine methyltransferase (CARM1/PRMT4 [PF3D7_0811500]). Indeed, PRMT inhibitors are active against *P. falciparum* parasites (121) and CARM1/PRMT4 is essential in asexual parasites (30), supporting the functional importance of these marks. Protein arginine methyltransferase inhibitors are seen as promising anticancer targets (122) and could be applied in the malaria context for gametocyte-targeting, transmission-blocking compounds.

The connectivity of histone PTMs in the *P. falciparum* histone code is associated with different developmental outcomes, similar to other eukaryotic systems requiring specialization, *e.g.*, embryogenesis and stem cell differentiation (123, 124). The prevailing heterochromatic mark, H3K9me3, is highly connected but not codependent on any other acetylation or methylation mark across all the life cycle stages. In the absence of quantified association between H3S10ph, as coexisting mark impairing HP1 binding to H3K9me3 (14) in Plasmodia, this indicates that H3K9me3 is likely singularly important to binding of HP1 to demarcate heterochromatin in *P. falciparum*. However, gametocytes additionally use other highly connected but independently acting repressive marks such as H3R40me1 and H3K14me2 to likely govern strategy-specific gene inactivation, as has been described for H3K36me2/3 inactivation of gene sets typically only required in asexual parasites (25). The observation that repressive marks are highly connected but usually show independence or mutual exclusivity to the partner PTMs questions the importance of repression in Plasmodia. The limited set of histone PTMs to enable effective transcriptional repression and induction of heterochromatin could be important to control transcript levels of gene sets, *e.g.*, virulence genes in asexual parasites (79) but are more important during gametocytogenesis. Additional mechanisms including RNA decay may be more influential to regulate transcript levels in general (125, 126).

Connectivity between PTMs in *P. falciparum* is important for coordinated function and activation of euchromatin. Silencing PTMs usually form independent heterochromatic domains (*e.g.*, H3K9me3), but activating marks are frequently found together. The majority of activating PTMs coordinate irrespective of life cycle stages, particularly H3K9acK14ac, and explains the euchromatic permissive nature of asexual parasites, enabled by coordinated binding of effector proteins. Novel combinations such as H3K4me3K23me1 and several combinations with H3R17me1 and H3R17me2 (H3K4me1, H3R8me2, H3K9me2) is pronounced during gametocytogenesis. In addition, during these stages of unique differentiation of *P. falciparum*, the parasite relies on specific and differentiated combinations, including the unique H3K27me1K36me1 and H3R17me2K18acK23ac combinations seen in only mature gametocytes. The connectivity and cross talk between histone PTMs are therefore essentially important to establish general euchromatic regions in the parasite’s genome across all stages. However, cross talk of activating marks is more prevalent in gametocytes and requires differentiation in marks used for euchromatin in these stages. This implies the use of specific histone combinations for activation of strategy-specific gene sets to mediate *P. falciparum* transmission.

The functional relevance of the connectivity of histone PTMs is underscored by evidence that the associated interacting proteins are also highly connected to include reader and writer proteins, “flavored” to specific marks (103). In this manner, the cross talk in the PTM combinations results in the recruitment of protein complexes to interpret the PTM combinations and allow changes to the chromatin structure. Indeed, we show that the unique codependent combination in mature gametocytes, H3K18acK23ac, jointly recruits the transcription factor AP2-G2, to initiate a SAGA-like complex containing GCN5/ADA2, flavored with K18acK23ac-specific effectors. Since codependency extends to the triple combination H3R17me2K18acK23ac, we provide evidence that this combination may indeed functionally associate with a gametocyte-specific SAGA-like complex to mediate stage-specific gene expression exclusively in mature gametocytes, as this combination has been demonstrated to induce transcriptional activation in cancer cells (97). The identification of AP2-G2 as an immediate binding partner on the H3K18acK23ac combination suggests that, in mature gametocytes, AP2-G2 may act as a transcriptional activator by targeting these histone PTM combinations. Investigations of the specific gene sets controlled by H3R17me2K18acK23ac and AP2-G2 in mature gametocytes are underway.

The histone code in *P. falciparum* is therefore diverse and dynamic to effect different combinations required for proliferation and differentiation. The complex nature of the combinations and changes in the identity of the combinations during stage transition point to this epigenetic level of regulation as a more important level of regulation that can be easily fine-tuned by variation in the combinations, particularly for gametocytes. With a limited set of effector proteins and only a core of ∼5 full effector protein complexes characterized (103), the intricacy of the histone code indicates that, indeed, combinations of histone PTMs provide the blueprint to ensure differentiation, specificity, and variation to control transcriptional activation of gene sets during *P. falciparum* development.

In conclusion, our study contributes a comprehensive catalog of histone PTM combinations and provides a foundation for further investigation of the increasingly intricate histone code of the *P. falciparum* parasite.

## Data Availability

The middle-down proteomics data generated in this study have been deposited in the Chorus database (https://chorusproject.org) and are accessible through project number 1721. All raw files from the ChIP-MS data are freely available on https://chorusproject.org at project no. 1730. The data analysis pipeline meets all MIAPE standards.

All proteomics data including annotated spectra of the modified peptides identified in this study are available *via* the ProteomeXchange Consortium (http://www.proteomexchange.org/) using the PRIDE partner repository (127), with data sets identifier PXD030181. The data analysis pipeline meets all MIAPE standards.

## Supplemental data

This article contains supplemental data.

## Conflict of interest

The authors declare that they have no conflict of interest with the contents of this article.
