# Supplementary material for: A Dynamic and Combinatorial Histone Code Drives Malaria Parasite Asexual and Sexual Development
Source: Mol Cell Proteomics. 2022 Jan 17;21(3):100199. doi: 10.1016/j.mcpro.2022.100199 (PMC8941266; doi:10.1016/j.mcpro.2022.100199)
Supplement: Supplemental Figures S1–S5 [file mmc1.pdf]

A dynamic and combinatorial histone code drives malaria parasite asexual and sexual development

Hilde von Grüning[1,2], Mariel Coradin[3,5], Mariel R. Mendoza[3], Janette Reader[1], Simone Sidoli[4], Benjamin A. Garcia[3], Lyn-Marie Birkholtz[1,2*]

[1] Department of Biochemistry, Genetics and Microbiology, University of Pretoria, Private bag X20, Hatfield, Pretoria, South Africa.

[2] Institute for Sustainable Malaria Control, University of Pretoria, Private bag X20, Hatfield, Pretoria, South Africa.

[3] Epigenetics Institute, Department of Biochemistry and Biophysics, Perelman School of Medicine, University of Pennsylvania, Philadelphia, PA 19104, USA.

[4] Department of Biochemistry, Albert Einstein College of Medicine, Bronx, NY 10461, USA.

Current address:

[5] Department of Molecular, Cellular, and Developmental Biology, University of Colorado Boulder, Boulder, CO 80309, USA.

[*]Corresponding author: L Birkholtz

Email: lbirkholtz@up.ac.za

Tel: +27 12 420 2479

Data provided:

- **Fig S1**: Protein yield, ETD dissociation scheme and biological replicate correlations as part of adapting middle-down proteomics for *P. falciparum* parasite analysis.
- **Fig S2**: Significant differences in histone PTM abundances between life cycle stages and histones H3 and H3.3.
- **Fig S3:** Co-existence frequencies of multiple PTMs in *P. falciparum*.
- **Fig S4:** The number of histone crosstalk combinations, the number of shared combinations between life cycle stages and a detailed list of the dynamic interplay scores observed.
- **Fig S5**: Validation of antibody specificity used for ChIP-MS experimentation, the distribution of intensity based absolute quantification values of identified proteins and number of proteins shared between other chromatin-based proteomic data sets.

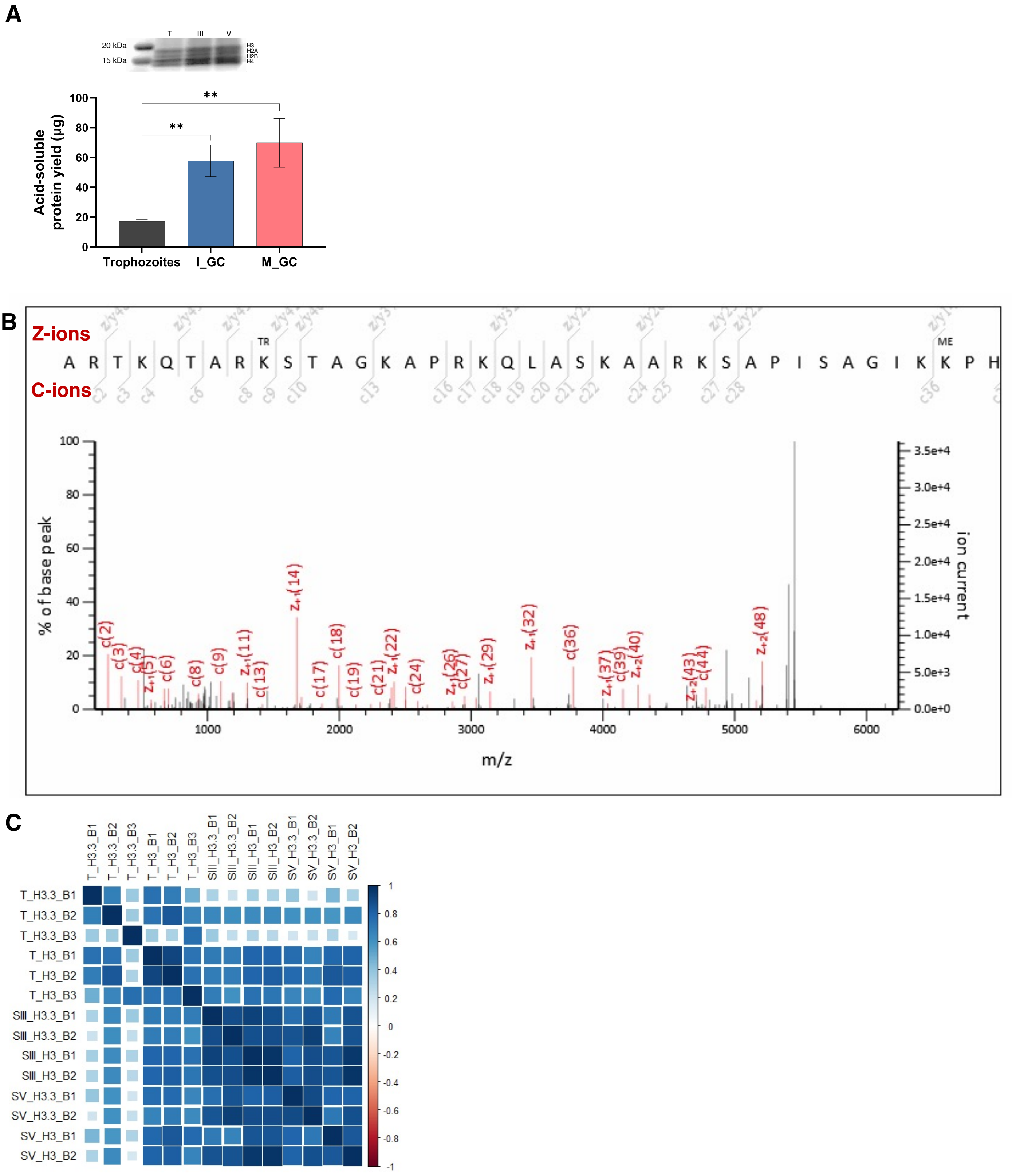

Figure S1. Adapting middle-down proteomics for *P. falciparum* parasites. (A) The acid soluble protein yield (µg) fractions for each developmental stage were determined (two-tailed equal variance t-test across the stages for each given PTM [***P*<0.01], with the average for trophozoites [n=3, mean ± SEM] and average for gametocytes [n=2, mean ± SD]). Histones refer to the isolated acid-soluble protein fraction containing the histone proteins and not to pure isolated histones. SDS-PAGE inset showing the histone fraction proteins, including bands representing histone H3, H2A, H2B and H4. **(B)** The dissociation scheme is shown for 35 amino acids of the same 50 amino acid peptide, notating for product c- and z-ions (amino and carboxy terminus of peptide, respectively). Base peak corresponds to the most abundant ion or the most intense peak in the spectrum (not in representative Mascot view, also referred to as the relative abundance). Both fragment and precursor *m/z* were measured in a linear ion trap. **(C)** Pearson correlation computation of the H3 and H3.3 samples of Trophozoites (T), immature (SIII) and mature stage gametocytes (SV).

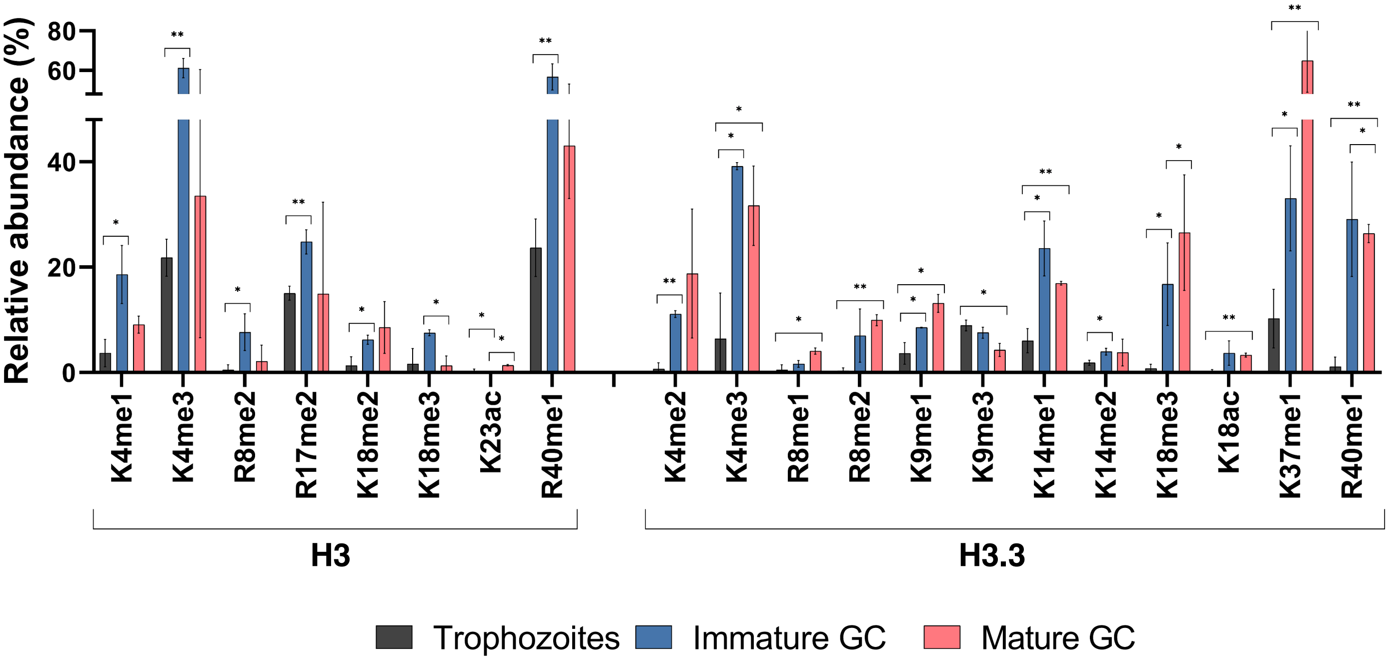

Figure S2. The relative abundances of histone H3 and H3.3 PTMs that indicated significant differences in relative abundances between trophozoites, immature gametocytes (GC) and mature gametocytes. A two-tailed equal variance t-test was performed across the stages for each given PTM (**P*<0.05; ***P*<0.01), for n=3 independent biological repeats (trophozoites) and n=2 for immature and mature gametocytes, mean ± SEM indicated.

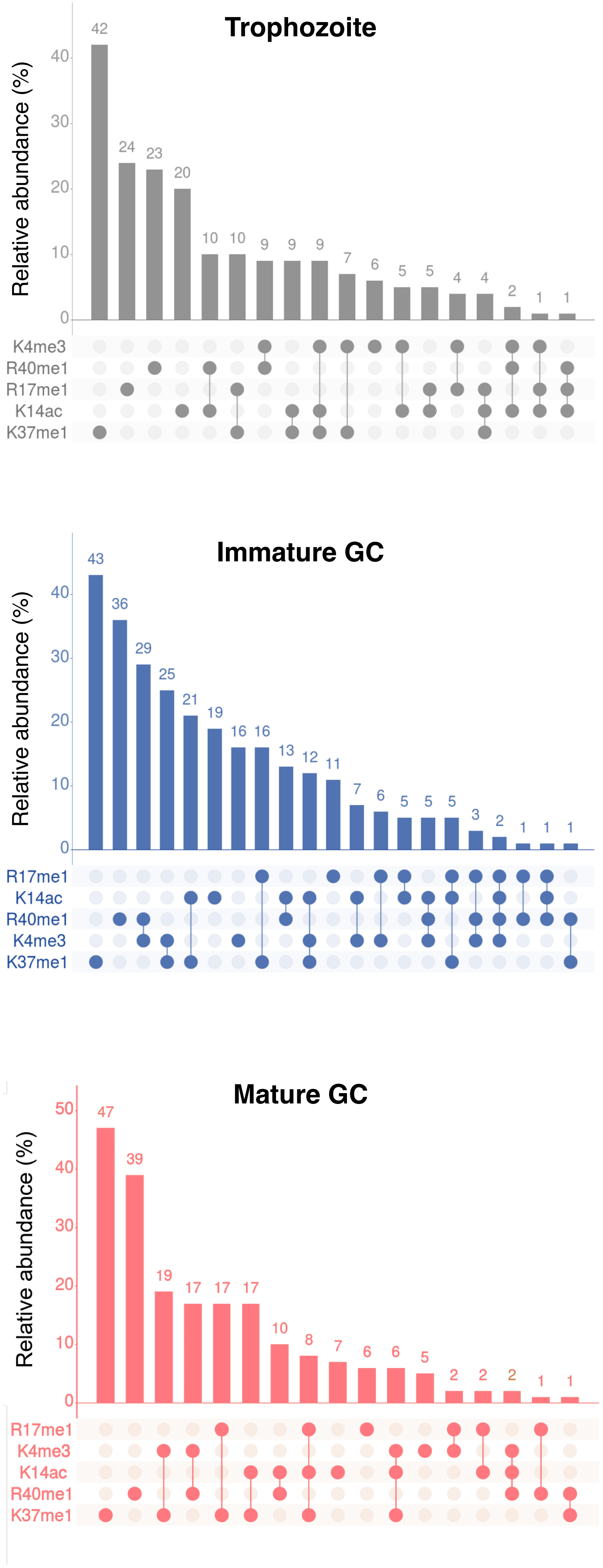

Figure S3. Co-existence frequencies of multiple PTMs in *P. falciparum*. UpSetR (https://jku-vds-lab.at/tools/upset/) plot showing the co-existence frequencies of multiple PTMs in trophozoites (top panel, grey), immature gametocytes (middle panel, blue) and mature gametocytes (bottom panel, pink).

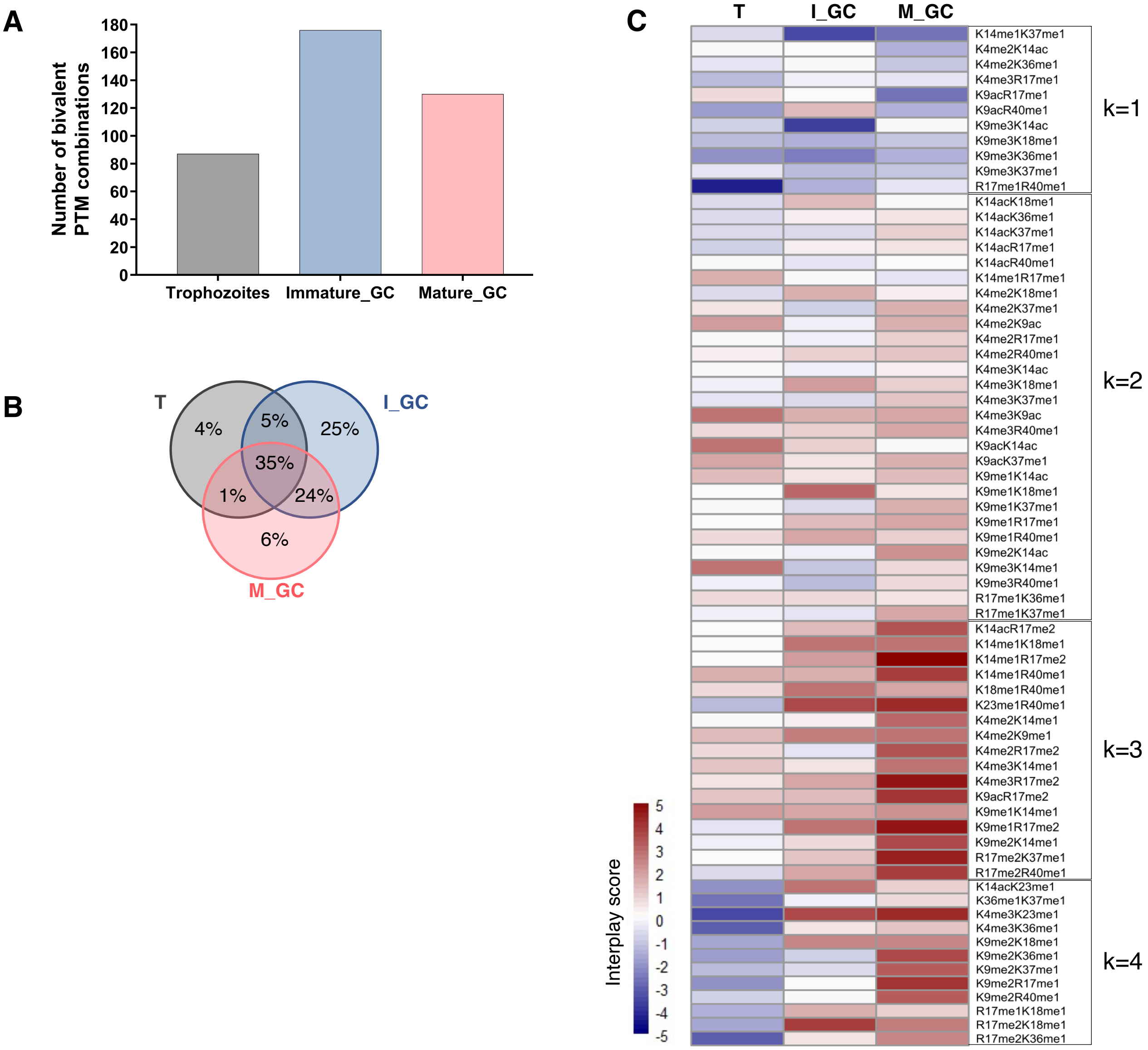

Figure S4. Histone modification crosstalk conserved across life cycle stages in *P. falciparum*.
(A) The number of bivalent crosstalk combinations characterized. **(B)** Venn diagram showing the overlap of bivalent combinations between trophozoites (T, grey), immature gametocytes (I_GC, blue) and mature gametocytes (M_GC, pink). **(C)** Interplay scores for PTM combinations consistently present in all life cycle stages analysed. Heatmap shows k-means clustering (complete, k=4) of the overlapping 35 % or 68 bivalent combinations and the respective interplay scores shared between all three stages. The average interplay scores are summarised for each cluster (cluster 1-4) across the life cycle stages.

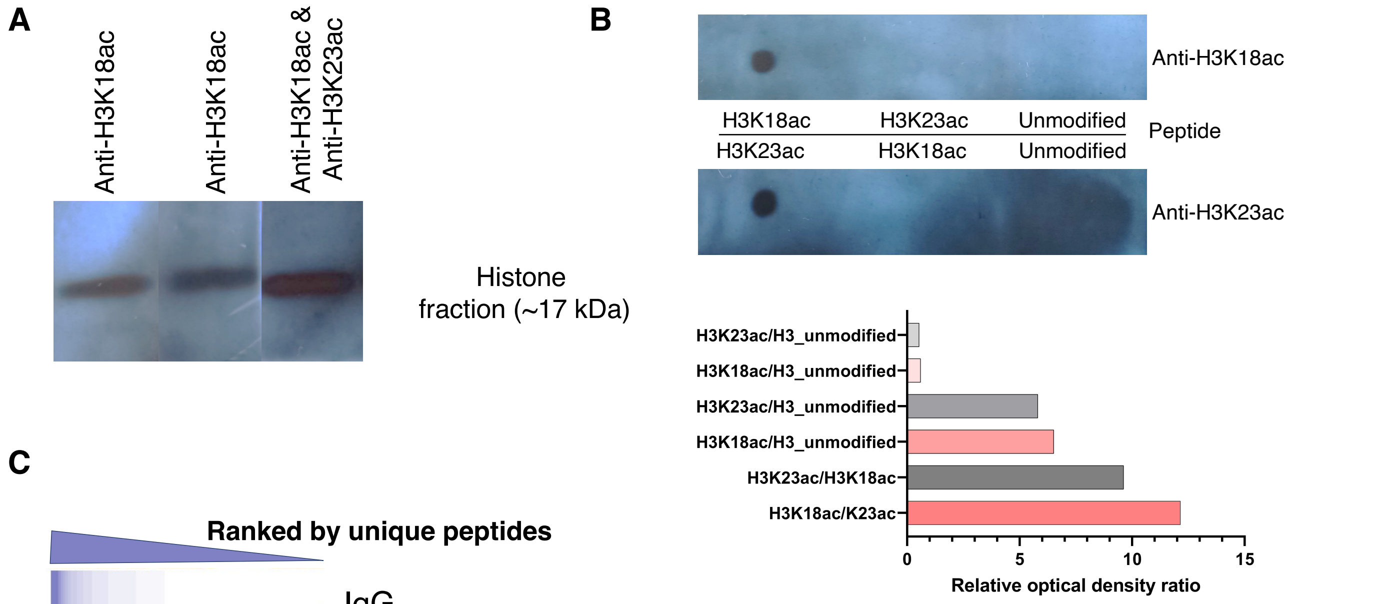

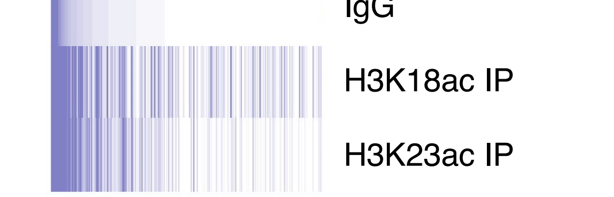

Peptide	Peptide Sequence	Length	Purity
H3K18ac	STAGKAPR{Lys-Ac}QLASKAAR	17	≥85%
H3K23ac	APRKQLAS{Lys-Ac}AARKSAPI	17	≥85%
Unmodified H3	STAGKAPRKQLASKAARKSAPI	22	≥85%

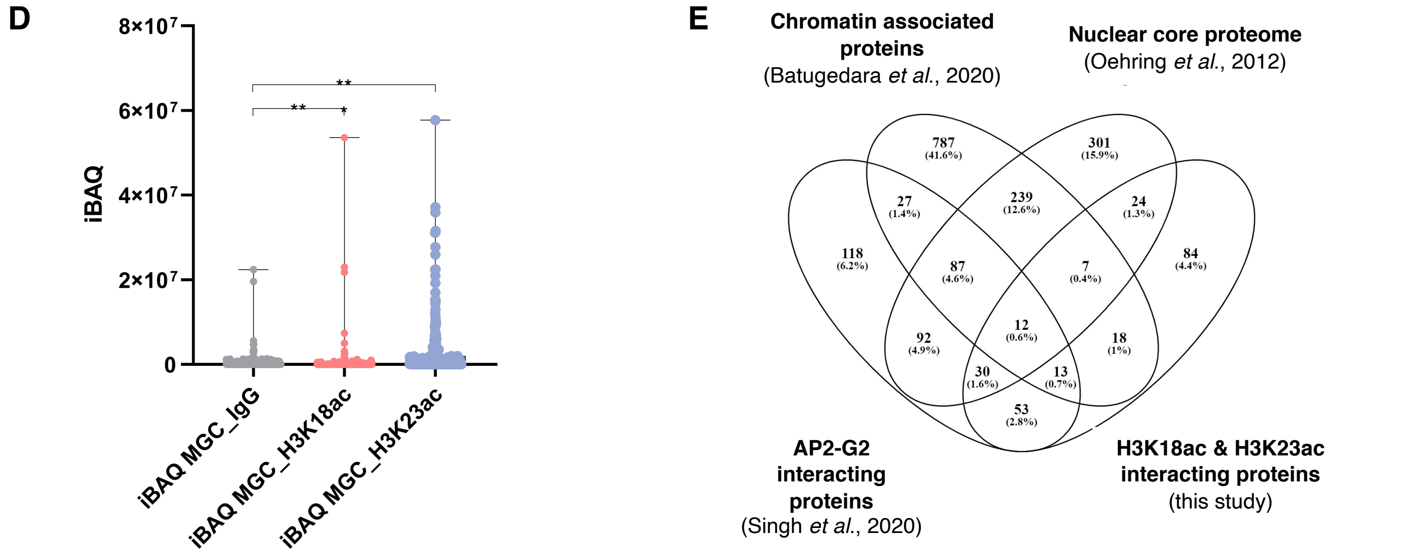

Figure S5. Validation of α-H3K18ac and α-H3K23ac antibodies to specifically detect *P. falciparum* histone H3 with the respective modifications. (A) The presence of both H3K18ac and H3K23ac marks were confirmed by western blot analysis, showing co-existence in mature stage gametocytes. **(B)** Dot blot analysis using unmodified (H3K18 and H3K23) and modified (H3K18ac, H3K23ac) synthetic histone peptides. Relative optical density was calculated to quantify the degree to which the antibodies detect each peptide. The H3K18ac and H3K23ac antibodies had 7.5- and 6-fold greater specificity towards their corresponding modified peptides compared to the unmodified peptides, respectively. The synthetic peptide sequence information is provided. **(C)** The distribution of detected unique proteins in the IgG, H3K18ac and H3K23ac ChIP-MS preparations (n=3, average per major identified protein). In all samples, the proteins were ranked according to their peptide counts. The presence of peptide counts for a protein in each preparation was marked with a blue line in the corresponding protein position. Proteins with lower abundance in the input were detected more often in the histone mark preparations than in the IgG preparation. **(D)** Distribution of the average iBAQ intensity values per protein identified for IgG, H3K18ac and H3K23ac sample preparations. **(E)** Venn diagram analysis of proteins identified in the H3K18ac and H3K23ac samples preparations with a positive $\log_2$ fold-change value above the IgG ChIP control and proteins identified in the AP2-G2 immunoprecipitation identified in Singh *et al.*, 2020, chromatin associated proteins from Batugedara *et al.*, 2019 and the nuclear proteome proteome (Oehring *et al.*, 2012).
